# Joint trajectories of online and offline social activity, cognitive decline, and frailty progression among middle-aged and older adults in China

**DOI:** 10.3389/ijph.2026.1610116

**Published:** 2026-10-14

**Authors:** Xueshan Sun, Danyi Sun, Zhongliang Zhou, Jiao Lu

**Affiliations:** School of Public Policy and Administration, Xi’an Jiaotong University, Xi’an, China

**Keywords:** cognitive decline, digital engagement, frailty progression, joint trajectories, social activity

## Abstract

**Objectives:**

To identify joint trajectories of online and offline social activity and examine their associations with cognitive decline and frailty progression among middle-aged and older adults in China.

**Methods:**

Data from 14,340 adults aged ≥45 years in CHARLS (2011–2018) were analyzed; outcome-specific samples included 10,323 participants for cognition and 13,960 for frailty. Group-based multi-trajectory modeling identified trajectories. Linear mixed-effects models estimated associations with annual changes in cognition and frailty.

**Results:**

Three trajectories were identified: persistently low social activity engagement (G1, 47.8%), offline social activity dominant (G2, 47.0%), and online social activity increasing (G3, 5.2%). Compared with G1, G2 showed slower cognitive decline (0.010 SD units/year, 95% CI [0.002, 0.018]) and frailty progression (−0.014 SD units/year, 95% CI [−0.020, −0.007]); G3 showed stronger associations for cognition (0.044 SD units/year, 95% CI [0.029, 0.060]) and frailty (−0.041 SD units/year, 95% CI [−0.055, −0.026]).

**Conclusion:**

The Offline Social Activity Dominant Group was associated with slower cognitive decline and frailty progression, with stronger associations for the Online Social Activity Increasing Group. Integrating online and offline social participation may support healthy aging.

## Introduction

Cognitive decline and frailty progression are growing global health challenges amid rapid population aging [1]. Cognitive decline represents a progressive deterioration in cognitive performance and is a core feature of dementia [2]. According to the World Alzheimer Report 2023, the number of people living with dementia worldwide is expected to rise from 55 million in 2019 to 139 million in 2050, while the associated annual costs are projected to increase from US$1.3 trillion per year in 2019 to US$2.8 trillion by 2030 [3]. Meanwhile, evidence increasingly shows a strong connection between frailty and cognitive impairment, as shared underlying mechanisms affect both domains [4]. Frailty, characterized by increased vulnerability to adverse health outcomes, has an estimated global incidence of 43.4 and 150.6 new cases per 1,000 person-years for frailty and prefrailty, respectively [5, 6]. Identifying scalable and modifiable behavioral factors that may help delay these aging-related processes has become an important public health priority.

A growing body of evidence suggests that social and lifestyle engagement may contribute to healthier cognitive and physical aging [3, 7–9]. Previous studies suggest that engagement in social and cognitively stimulating activities may contribute to better cognitive and physical health among middle-aged and older adults [8]. Social participation theory suggests these social activities provide psychosocial resources that reduce stress and depression, known risk factors for cognitive decline and frailty [10, 11]. This aligns with the “use it or lose it” principle, wherein sustained activity helps maintain neural and physiological function [12]. In parallel with traditional forms of social participation, digital engagement has become increasingly relevant for middle-aged and older adults [13]. Online engagement may provide opportunities for cognitive stimulation, social connection, health information access, and self-management, with potential relevance to cognitive and physical health [14, 15]. In rapidly digitalizing societies, online engagement may therefore represent an emerging and potentially scalable pathway to support healthy aging. Taken together, existing evidence suggests that both online and offline social activity engagement may contribute to cognitive and physical health; however, how these two forms of engagement jointly develop over time remains less well understood.

Several important gaps remain. First, although longitudinal studies have examined trajectories of social activity and have considered Internet use together with social participation among middle-aged and older adults [16, 17], little is known about the joint longitudinal trajectories of online and offline social activity engagement. Second, limited evidence has examined how these joint trajectories are simultaneously associated with cognitive decline and frailty progression over time. Third, evidence on these associations remains particularly limited in large population-based cohorts of middle-aged and older adults in China. Addressing these gaps is important for developing public health strategies that promote multidimensional social activity engagement and reduce inequalities in healthy aging.

Therefore, using seven-year longitudinal data from the China Health and Retirement Longitudinal Study (CHARLS), this study aimed to identify joint trajectories of online and offline social activity engagement among middle-aged and older Chinese adults and to examine their longitudinal associations with cognitive decline and frailty progression. By clarifying how distinct joint trajectories of online and offline social activity engagement are associated with cognitive and physical aging, this study may provide evidence to support the development of public health interventions and policies that promote social activity, digital engagement, and healthy aging in rapidly aging populations.

## Methods

### Study design and participants

This longitudinal cohort study used data from CHARLS, a nationally representative survey primarily targeting Chinese adults aged 45 years and older. Spouses of age-eligible respondents were also interviewed regardless of their own age [18]. CHARLS enrolled more than 17,000 participants across 28 provinces at baseline in 2011–2012, with follow-up assessments conducted every 2 years through face-to-face computer-assisted interviews. Further details have been reported elsewhere [18]. All procedures performed in this study involving human participants were in accordance with the ethical standards of the Peking University Institutional Review Board (IRB00001052-11015) and with the 1964 Helsinki declaration and its amendments or comparable ethical standards.

This analysis included four waves (2011–2018), with 2011 defined as baseline. Data were drawn from both the Harmonized CHARLS Version D and the original CHARLS databases. Of 17,708 baseline participants, 777 were excluded due to age <45 years or missing age information, and 2,591 were further excluded due to missing baseline online and offline social activity data or lack of follow-up assessments. A total of 14,340 participants were included in the trajectory analysis. Outcome-specific analytic samples were then constructed based on availability of repeated outcome measures [19], excluding participants with diagnosed memory-related diseases, missing baseline outcome data, or no follow-up assessments. This resulted in analytic samples of 10,323 for cognition, 13,960 for frailty, 12,070 for memory, 12,926 for orientation, and 11,930 for executive function (Supplementary Figure SA1). Compared with included participants, those excluded from the cognitive analytic sample were generally older, more likely to be female, less educated, and had poorer baseline cognition and higher frailty, whereas baseline characteristics were broadly comparable in the frailty analytic sample (Supplementary Table SA1).

### Measurements

#### Independent variable

The independent variable was the joint trajectory group of online and offline social activity engagement over the seven-year follow-up. Offline social activity was assessed using six self-reported social activities in the past month: interacting with friends; playing mahjong, chess, or cards, or attending a community club; participating in sport, social, or other clubs; taking part in community-related organizations; volunteering or charity work; and attending educational or training courses [20, 21]. Each activity was coded as 1 for participation and 0 otherwise, and summed to create an offline activity score ranging from 0 to 6. Online activity was defined as reporting internet use as a social activity or stock investment during the past month. Internet use was measured by a binary question asking whether participants had used the internet as a social activity in the last month. Stock investment was included in the primary online activity definition because it may involve digital financial engagement. Given that it was not necessarily internet-based, we conducted a sensitivity analysis defining online social activity using internet use only. Participants reporting either activity were classified as engaging in online activity. Joint trajectories of online and offline social activity engagement were identified, and the final model identified three groups: G1, Persistently Low Social Activity Engagement Group; G2, Offline Social Activity Dominant Group; and G3, Online Social Activity Increasing Group (Figure 1; Supplementary Tables SA2–SA4).

**FIGURE 1:**
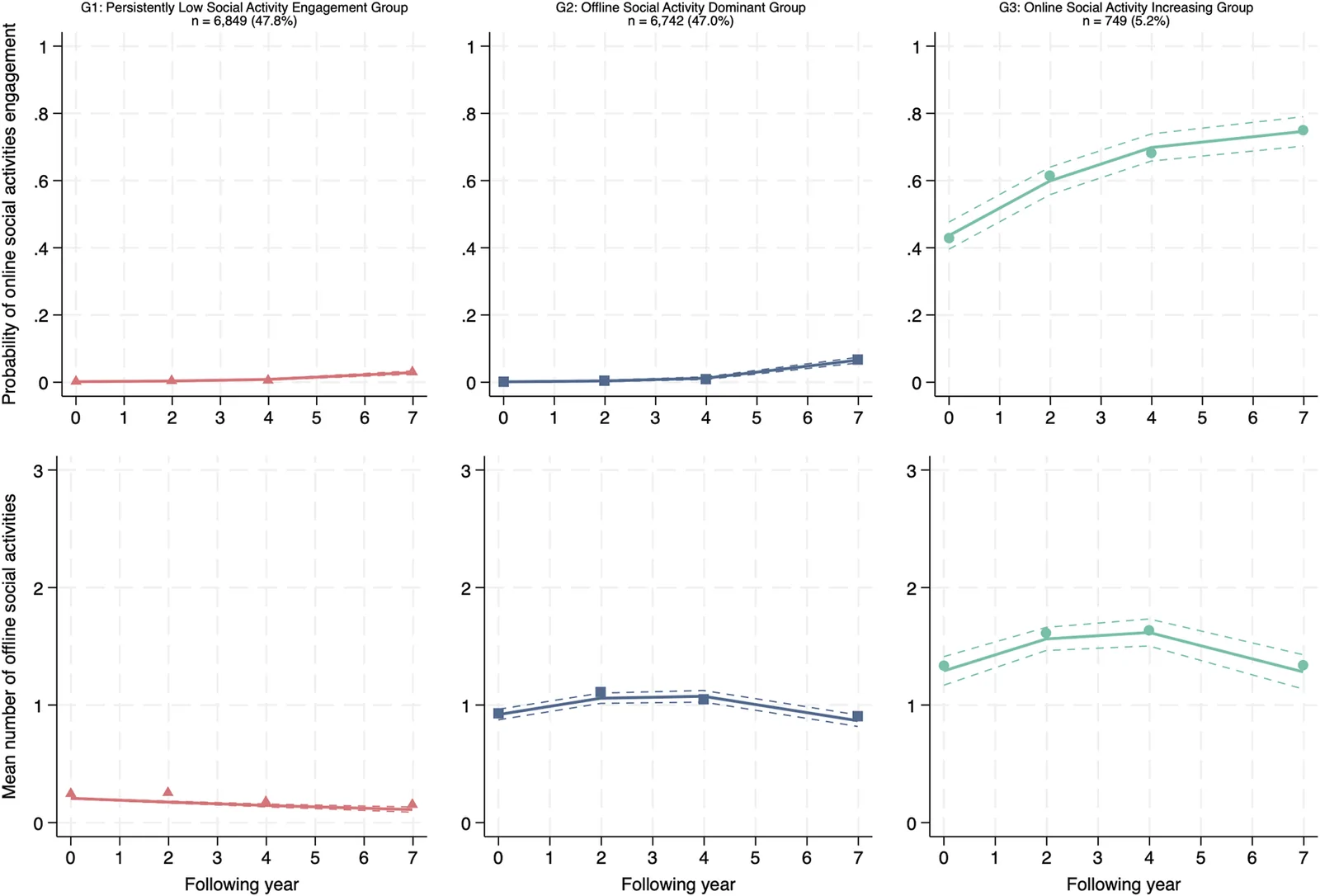
Joint trajectories of online–offline social activity engagement (China, 2011–2018). Note: Columns represent the three identified trajectory groups: G1 (n = 6,849, 47.8%), G2 (n = 6,742, 47.0%), and G3 (n = 749, 5.2%). Solid lines represent model-estimated trajectories, dashed lines indicate the corresponding 95% confidence intervals, and points represent the observed proportions of online social activity engagement and observed mean numbers of offline social activities at each wave. Abbreviations: G1, Persistently Low Social Activity Engagement Group; G2, Offline Social Activity Dominant Group; G3, Online Social Activity Increasing Group.

#### Dependent variables

The primary outcomes were global cognitive function and physical frailty, and the secondary outcomes were memory, executive function, and orientation. Cognitive function was measured across three domains: memory, executive function and orientation [22, 23]. Memory was evaluated by immediate and delayed recall of 10 words, with one point awarded for each correctly recalled word (score range: 0–20). Executive function was measured using the serial-7 subtraction test (score range: 0–5). Orientation was assessed by asking participants to report the current date and day of the week (score range: 0–4). Memory, executive function, and orientation scores were first standardized using their respective baseline means and SDs, $Z_{dit}=\left(X_{dit}-{\bar{X}}_{d,baseline}\right)/{SD}_{d,baseline}$ , where $X_{dit}$ represents the score for cognitive domain *d* of participant *i* at wave *t,* ${\bar{X}}_{d,baseline}$ and ${SD}_{d,baseline}$ denote the baseline mean and SD of domain *d*, respectively. The three domain-specific Z-scores were then averaged to create a composite cognition score, which was subsequently standardized using its baseline mean and SD to generate the global cognitive Z-score used in the longitudinal models. Consequently, a Z-score of −1 at a follow-up indicates that global cognitive performance was one SD below the baseline mean. Changes in global cognition over time were estimated using mixed-effects models and expressed in SD units per year [22, 23].

Physical frailty was measured using a frailty index based on accumulated health deficits [24]. To avoid potential criterion contamination with cognitive and psychological outcomes, memory-related disease and emotional, nervous, or psychiatric problems were excluded from the FI construction. The index included 34 deficits covering chronic diseases, physical function, mobility, and sensory impairments (Supplementary Table SA5). For ADL, IADL, and mobility items, “no difficulty” was coded as 0, whereas “difficulty but can still do it”, “difficulty and need help”, and “cannot do it” were coded as 1, which was consistent with the Harmonized CHARLS Version D and prior studies [25, 26]. Each deficit was dichotomized or scaled from 0 to 1, and the frailty index was calculated as the ratio of accumulated deficits to total deficits [24]. The index was multiplied by 100 and standardized as a Z-score.

#### Control variables

Control variables were assessed at baseline and selected based on prior evidence. These included age, gender, educational level, marital status, residence, *per capita* annual household income, medical insurance coverage, current employment status, physical exercise, smoking, and alcohol consumption. Age was treated as a continuous variable. Educational level was categorized as less than high school, high school or equivalent, and college or above; *per capita* annual household income was classified as below the median or at or above the median based on the median of the analytic sample. Other covariates were coded as binary variables. Baseline levels of the corresponding outcomes were additionally adjusted for in the longitudinal mixed-effects models.

### Statistical analysis

Data analysis was conducted in June 2026. Baseline characteristics of participants were summarized by joint trajectory group. Categorical variables were described as frequencies and percentages, ordinal variables as medians with interquartile ranges (IQRs), and continuous variables as means with standard deviations (SDs). Between-group differences were assessed using chi-square tests for categorical variables, the Kruskal-Wallis test for ordinal variables, and analysis of variance (ANOVA) for continuous variables.

Joint trajectories of online and offline social activity engagement were identified using group-based multi-trajectory modeling. Online social activity was modeled using a logit model, whereas the offline social activity score (0–6) was modeled using a censored normal model. Candidate models with two to four groups and different polynomial orders were compared, and the final three-group model used the 112–122 specification. Model selection considered BIC, AIC, AvePP, OCC, entropy, group size, and substantive interpretability. Default starting-value and convergence settings of the traj procedure were used, with detailed diagnostics reported in Supplementary Tables SA2–SA4.

Linear mixed models were used to examine the longitudinal associations between joint activity trajectory groups and changes in cognition and frailty [20, 23, 27]. The fixed effects included trajectory group, continuous time in years since baseline, and the interaction between trajectory group and time. Time was coded as 0 at baseline, and no additional centering was applied; therefore, model intercepts represent estimated baseline outcome levels. Random intercepts and random slopes for time were included to account for within-participant correlation over repeated measurements. In these models, a positive interaction coefficient indicates slower cognitive decline but faster frailty progression, whereas a negative interaction coefficient indicates faster cognitive decline but slower frailty progression [23]. Because social activity trajectories and changes in cognitive function and frailty were assessed over the same 2011–2018 observation period, the trajectory group × time interactions were interpreted as joint longitudinal associations rather than evidence that activity trajectories temporally preceded subsequent changes in cognition or frailty.

Heterogeneity analyses were conducted to examine whether key population characteristics modified the longitudinal associations of interest. Analyses were stratified by gender (male vs. female), age (<65 vs. ≥65 years), residence (urban vs. rural), and *per capita* annual household income (below the median vs. at or above the median). Stratified linear mixed-effects models were fitted within each subgroup to describe subgroup-specific estimates. Formal effect modification was evaluated using trajectory group × time × heterogeneity-variable three-way interaction terms, with omnibus Wald tests jointly assessing the relevant three-way interaction coefficients. Exact interaction P values were reported. Differences in statistical significance between subgroup-specific estimates were not interpreted as evidence of heterogeneity. These heterogeneity analyses were considered exploratory.

Sensitivity analyses assessed the robustness of the findings. First, raw cognitive and frailty scores were used instead of Z-scores. Second, baseline CESD-10 scores were additionally adjusted for in a sensitivity analysis, as depressive symptoms may partly lie on the pathway linking social activity with cognitive and physical health and were therefore not included in the primary covariate set. Third, to further reduce the potential influence of reverse causation beyond excluding participants with baseline memory-related diseases, we additionally excluded participants with baseline cognitive impairment or frailty, then re-estimated trajectory groups and refitted the mixed-effects models. Cognitive impairment was defined as a global cognitive score more than 1 SD below the age-standardized mean [28], and frailty was defined as a frailty index ≥25 [29]. Fourth, to address potential misclassification from stock investment, we redefined online social activity using internet use alone, re-estimated the trajectory groups, and refitted the cognition and frailty models. Fifth, we reconstructed the FI by coding only “cannot do it” as a deficit for ADL, IADL, and mobility items and refitted the frailty model using the alternative FI and its corresponding baseline value. All analyses were performed using Stata SE 16.0 software (Stata Corp, College Station, Texas, United States). Two-sided p-values <0.05 were considered statistically significant.

## Results

### Baseline characteristics by joint trajectory groups

Three distinct joint trajectories of online and offline social activity engagement were identified: G1, the Persistently Low Social Activity Engagement Group (n = 6,849, 47.8%), characterized by persistently low engagement in both online and offline social activities; G2, the Offline Social Activity Dominant Group (n = 6,742, 47.0%), characterized by participation in a relatively greater number of offline activity types but limited online activity engagement; and G3, the Online Social Activity Increasing Group (n = 749, 5.2%), characterized by increasing online activity engagement combined with participation in a relatively greater number of offline activity types over time (Figure 1).

Baseline characteristics of the 14,340 participants are presented by joint trajectory group. The mean age was 58.7 years (SD = 9.3), and 47.5% were male. Participants reported an average of 0.6 offline activity types (SD = 0.8), while 2.6% reported any online activity engagement. Most participants had less than a high school education (88.4%), were married (88.0%), and lived in rural areas (61.4%). Other baseline characteristics and baseline cognitive and frailty measures are summarized in Table 1. Most baseline characteristics differed across trajectory groups, whereas alcohol consumption did not (Table 1).

**TABLE 1:** Baseline characteristics of participants by joint trajectory group (China, 2011–2018).

Variables	Overall	G1	G2	G3	p
Number	14,340 (100.0%)	6,849 (47.8%)	6,742 (47.0%)	749 (5.2%)	​
Age, years, mean (SD)	58.7 (9.3)	59.7 (9.3)	58.3 (9.1)	53.3 (7.3)	<0.001
Gender	​	​	​	​	<0.001
Male, n (%)	6,810 (47.5%)	3,244 (47.4%)	3,146 (46.7%)	420 (56.1%)	​
Female, n (%)	7,530 (52.5%)	3,605 (52.6%)	3,596 (53.3%)	329 (43.9%)	​
Education level	​	​	​	​	<0.001
Less than high school, n (%)	12,683 (88.4%)	6,407 (93.5%)	5,967 (88.5%)	309 (41.3%)	​
High school or equivalent, n (%)	1,408 (9.8%)	399 (5.8%)	688 (10.2%)	321 (42.9%)	​
College and higher, n (%)	249 (1.7%)	43 (0.6%)	87 (1.3%)	119 (15.9%)	​
Marital status	​	​	​	​	<0.001
Married, n (%)	12,613 (88.0%)	6,026 (88.0%)	5,893 (87.4%)	694 (92.7%)	​
Residence	​	​	​	​	<0.001
Rural, n (%)	8,801 (61.4%)	4,677 (68.3%)	4,008 (59.4%)	116 (15.5%)	​
Per capita annual household income	​	​	​	​	<0.001
Below the median, n (%)	7,157 (50.0%)	3,910 (57.2%)	3,162 (47.0%)	85 (11.3%)	​
At or above the median, n (%)	7,166 (50.0%)	2,931 (42.8%)	3,571 (53.0%)	664 (88.7%)	​
Medical insurance coverage	​	​	​	​	0.002
Insured, n (%)	13,358 (93.2%)	6,328 (92.4%)	6,323 (93.8%)	707 (94.4%)	​
Current employment status	​	​	​	​	<0.001
Currently working, n (%)	9,089 (64.4%)	4,454 (66.2%)	4,198 (63.1%)	437 (59.2%)	​
Physical exercise	​	​	​	​	0.034
Yes, n (%)	4,017 (28.0%)	1,924 (28.1%)	1,914 (28.4%)	179 (23.9%)	​
Alcohol consumption	​	​	​	​	0.703
Yes, n (%)	2,353 (16.4%)	1,106 (16.1%)	1,120 (16.6%)	127 (17.0%)	​
Current smoking	​	​	​	​	0.015
Yes, n (%)	4,397 (30.7%)	2,021 (29.5%)	2,145 (31.8%)	231 (30.8%)	​
Number of offline social activities, mean (SD)	0.6 (0.8)	0.3 (0.5)	0.9 (0.8)	1.4 (1.1)	<0.001
Online social activities engagement, n (%)	374 (2.6%)	16 (0.2%)	8 (0.1%)	350 (46.7%)	<0.001
Cognition score, mean (SD)	14.3 (4.5)	13.4 (4.4)	14.6 (4.3)	18.2 (3.4)	<0.001
Memory function score, mean (SD)	7.2 (3.3)	6.7 (3.2)	7.5 (3.3)	10.0 (3.1)	<0.001
Orientation score, median (IQR)	3 (2–4)	3 (2–4)	3 (3–4)	4 (4–4)	<0.001
Executive function score, median (IQR)	4 (2–5)	4 (2–5)	4 (3–5)	5 (4–5)	<0.001
Frailty index, mean (SD)	13.2 (11.9)	14.8 (12.9)	12.4 (10.8)	7.2 (7.2)	<0.001

Abbreviations*: SD*, standard deviation; G1, Persistently Low Social Activity Engagement Group; G2, Offline Social Activity Dominant Group; G3, Online Social Activity Increasing Group. *P* values are based on chi-square tests for categorical variables, Kruskal-Wallis tests for ordinal variables, and ANOVA for continuous variables. The global cognition score presented in Table 1 is the raw score for descriptive purposes, whereas the standardized global cognitive Z-score was used in the longitudinal models. Percentages were calculated using participants with non-missing data for the corresponding variable as the denominator.

### Joint trajectories and global cognitive decline and frailty progression

Longitudinal analyses revealed distinct patterns of cognitive change across joint trajectory groups. Global cognition declined significantly over time (coefficient = −0.055, 95% CI [-0.061, −0.049], p < 0.001). Compared with the Persistently Low Social Activity Engagement Group, the Offline Social Activity Dominant Group showed a modestly slower decline in global cognition (G2 × time: coefficient = 0.010, 95% CI [0.002, 0.018], p = 0.011). The Online Social Activity Increasing Group showed a stronger attenuation of cognitive decline (G3 × time: coefficient = 0.044, 95% CI [0.029, 0.060], p < 0.001). Frailty increased significantly over time (coefficient = 0.095, 95% CI [0.091, 0.100], p < 0.001). This progression was significantly modified by joint trajectories. Compared with the Persistently Low Social Activity Engagement Group, both the Offline Social Activity Dominant Group (G2 × Time: coefficient = −0.014, 95% CI [−0.020, −0.007], p < 0.001) and the Online Social Activity Increasing Group (G3 × Time: coefficient = −0.041, 95% CI [−0.055, −0.026], p < 0.001) experienced slower frailty progression over time (Figure 2; Table 2; Supplementary Table SA6).

**FIGURE 2:**
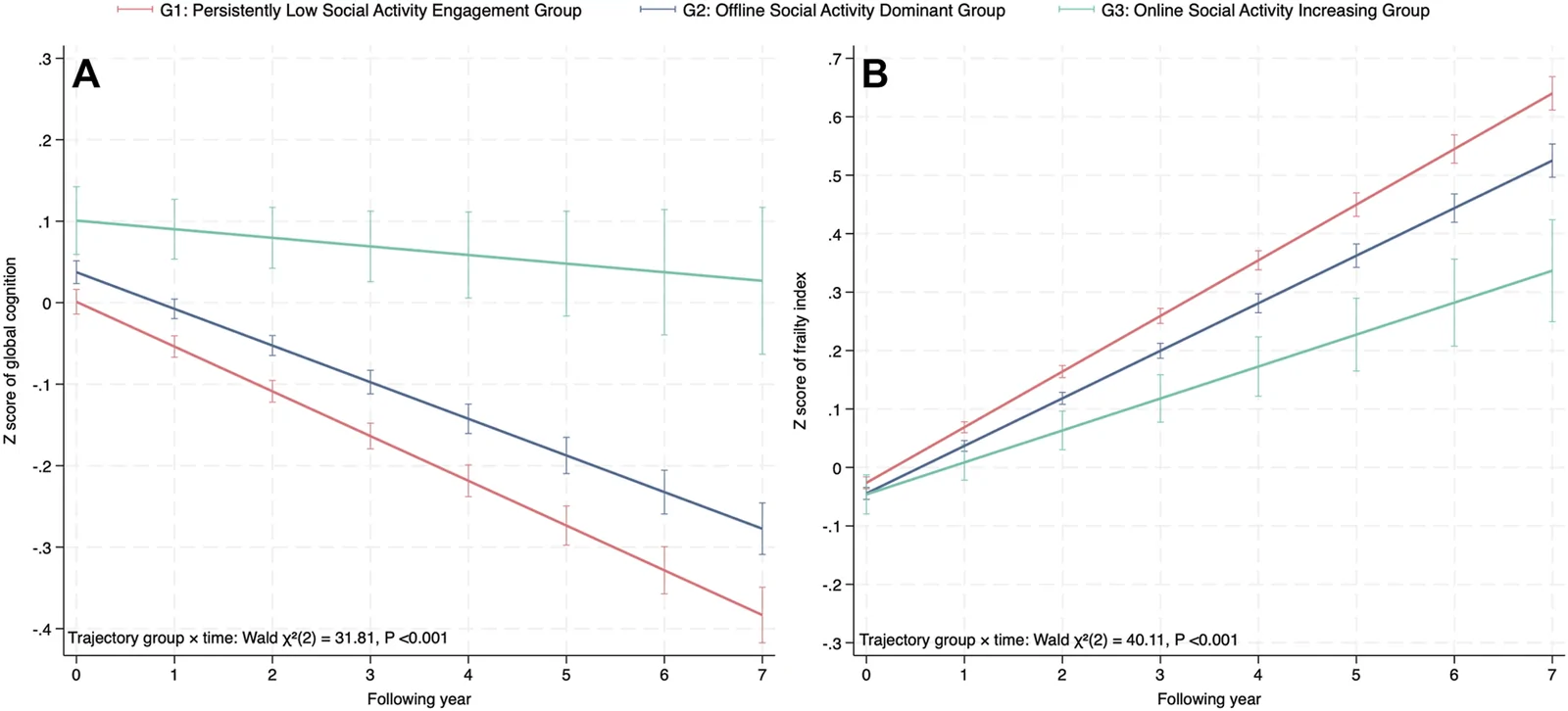
Trajectories of cognitive decline and frailty progression by joint online–offline social activity trajectory groups (China, 2011–2018). Note: Lines represent adjusted marginal predictions from mixed-effects models, and error bars indicate 95% confidence intervals. Predictions were averaged over the observed distribution of covariates. Models were adjusted for baseline age, gender, educational level, marital status, residence, *per capita* annual household income, medical insurance coverage, current employment status, physical exercise, alcohol consumption, smoking status, and baseline levels of the corresponding outcomes. The omnibus trajectory group × time interaction was assessed using a Wald test. Abbreviations: G1, Persistently Low Social Activity Engagement Group; G2, Offline Social Activity Dominant Group; G3, Online Social Activity Increasing Group. **(A)** Cognitive decline, **(B)** frailty progression.

**TABLE 2:** Longitudinal associations of joint online–offline social activity trajectory groups with changes in global cognition and frailty (China, 2011–2018).

Variables	Cognitive decline		Frailty progression	
	Coef [95% CI]	p	Coef [95% CI]	p
Time, years	−0.055 [-0.061, −0.049]	<0.001	0.095 [0.091, 0.100]	<0.001
Joint online–offline social activity trajectory groups				
G1	Reference	​	Reference	​
G2	0.036 [0.016, 0.057]	0.001	−0.018 [-0.033, −0.003]	0.016
G3	0.100 [0.055, 0.145]	<0.001	−0.020 [-0.055, 0.016]	0.278
Joint online–offline social activity trajectory groups × time				
G1 × time	Reference	​	Reference	​
G2 × time	0.010 [0.002, 0.018]	0.011	−0.014 [-0.020, −0.007]	<0.001
G3 × time	0.044 [0.029, 0.060]	<0.001	−0.041 [-0.055, −0.026]	<0.001

Models were adjusted for baseline age, gender, educational level, marital status, residence, *per capita* annual household income, medical insurance coverage, current employment status, physical exercise, alcohol consumption, smoking status, and baseline levels of the corresponding outcomes (z-scores). Abbreviations: Coef, coefficient; CI, confidence interval; G1, Persistently Low Social Activity Engagement Group; G2, Offline Social Activity Dominant Group; G3, Online Social Activity Increasing Group.

### Joint trajectories and domain-specific cognitive decline

Domain-specific analyses further showed significant declines over time in memory, orientation, and executive function (Table 3). The interaction patterns of joint activity trajectories varied across cognitive domains. Compared with the Persistently Low Social Activity Engagement Group, the Offline Social Activity Dominant Group showed a significantly slower decline in memory function (G2 × Time: coefficient = 0.022, 95% CI [0.014, 0.030], p < 0.001), whereas its interactions with time were not significant for orientation or executive function. The Online Social Activity Increasing Group showed the strongest attenuation of memory decline (G3 × Time: coefficient = 0.073, 95% CI [0.055, 0.090], p < 0.001) and also experienced a slower decline in executive function (G3 × Time: coefficient = 0.016, 95% CI [0.001, 0.031], p = 0.032). No significant trajectory-by-time interactions were observed for orientation function. Associations were most evident for memory function (Figure 3; Table 3; Supplementary Table SA7).

**TABLE 3:** Longitudinal associations of joint online–offline social activity trajectory groups with changes in cognitive subdomains (China, 2011–2018).

Variables	Memory function		Orientation function		Executive function	
	Coef [95% CI]	p	Coef [95% CI]	p	Coef [95% CI]	p
Time, years	−0.050 [-0.056, −0.044]	<0.001	−0.034 [-0.039, −0.029]	<0.001	−0.035 [-0.040, −0.029]	<0.001
Joint online–offline social activity trajectory groups						
G1	Reference	​	Reference	​	Reference	​
G2	0.034 [0.013, 0.055]	0.002	0.046 [0.025, 0.066]	<0.001	0.038 [0.019, 0.057]	<0.001
G3	0.096 [0.047, 0.144]	<0.001	0.083 [0.035, 0.130]	0.001	0.086 [0.044, 0.129]	<0.001
Joint online–offline social activity trajectory groups × time						
G1 × time	Reference	​	Reference	​	Reference	​
G2 × time	0.022 [0.014, 0.030]	<0.001	0.004 [-0.003, 0.011]	0.243	−0.001 [-0.008, 0.007]	0.882
G3 × time	0.073 [0.055, 0.090]	<0.001	0.011 [-0.004, 0.027]	0.145	0.016 [0.001, 0.031]	0.032

Models were adjusted for baseline age, gender, educational level, marital status, residence, *per capita* annual household income, medical insurance coverage, current employment status, physical exercise, alcohol consumption, smoking status, and baseline levels of the corresponding outcomes (z-scores). Abbreviations: Coef, coefficient; CI, confidence interval; G1, Persistently Low Social Activity Engagement Group; G2, Offline Social Activity Dominant Group; G3, Online Social Activity Increasing Group.

**FIGURE 3:**
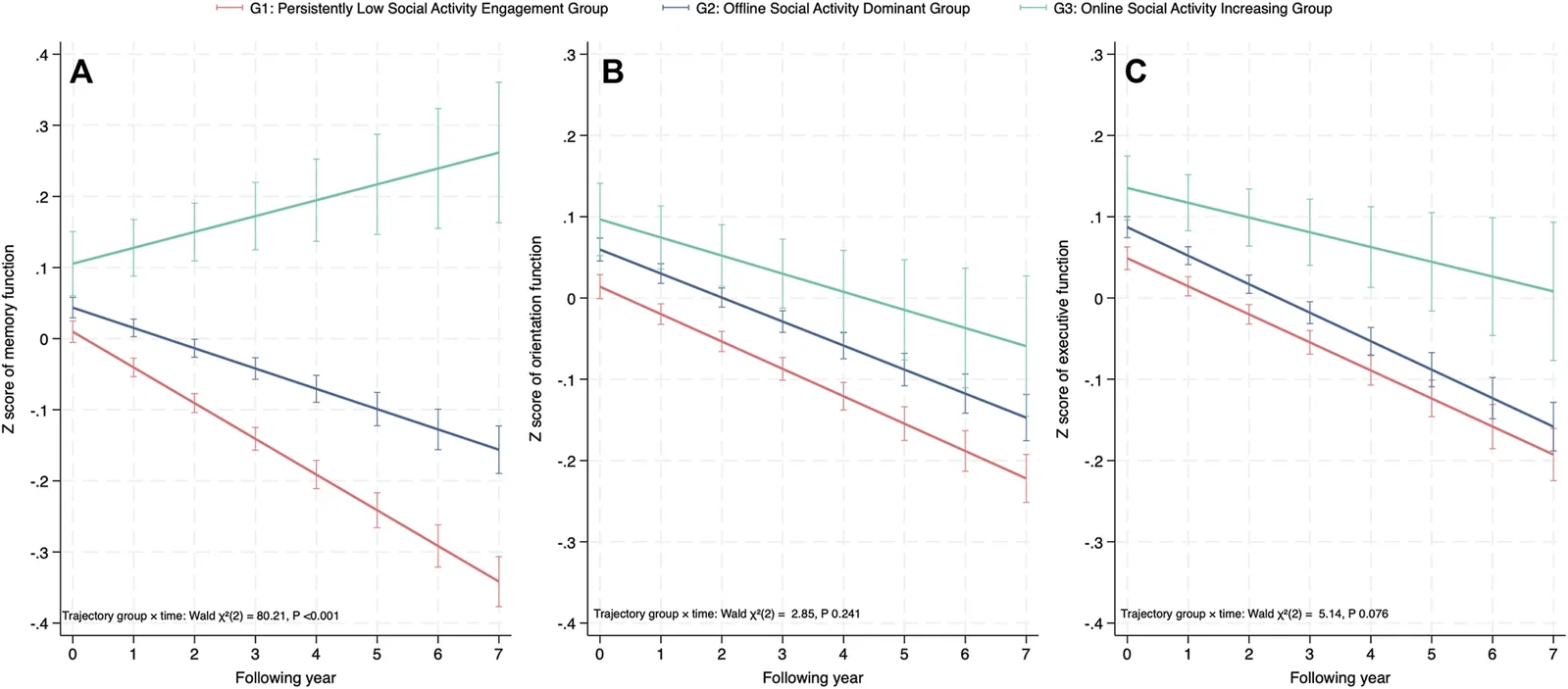
Trajectories of decline in three cognitive subdomains by joint online–offline social activity trajectory groups (China, 2011–2018). Note: Lines represent adjusted marginal predictions from mixed-effects models, and error bars indicate 95% confidence intervals. Predictions were averaged over the observed distribution of covariates. Models were adjusted for baseline age, gender, educational level, marital status, residence, *per capita* annual household income, medical insurance coverage, current employment status, physical exercise, alcohol consumption, smoking status, and baseline levels of the corresponding outcomes. The omnibus trajectory group × time interaction was assessed using a Wald test. Abbreviations: G1, Persistently Low Social Activity Engagement Group; G2, Offline Social Activity Dominant Group; G3, Online Social Activity Increasing Group. **(A)** Memory function, **(B)** orientation function, **(C)** executive function.

### Heterogeneity analysis results

Formal three-way interaction analyses showed no statistically significant evidence of effect modification by gender, age, residence, or *per capita* annual household income for either cognition or frailty. For cognition, the omnibus P values for interaction were 0.841 for gender, 0.562 for age, 0.736 for residence, and 0.815 for *per capita* annual household income. For frailty, the corresponding omnibus P values were 0.738, 0.489, 0.511, and 0.763, respectively (Supplementary Table SA8). Subgroup-specific estimates with 95% confidence intervals, exact P values, and sample sizes are presented in Supplementary Tables SA9, SA10. Differences in statistical significance between subgroup-specific estimates were not interpreted as evidence of effect modification. Because some subgroup-specific estimates, particularly those involving G3, were based on relatively small numbers of participants and had wider confidence intervals, these exploratory findings should be interpreted cautiously.

### Sensitivity analysis results

Across sensitivity analyses, the associations of the Offline Social Activity Dominant Group (G2) and the Online Social Activity Increasing Group (G3) with slower cognitive decline and frailty progression remained robust. First, using raw instead of Z-standardized scores, the G2 × Time and G3 × Time interactions remained significant for both outcomes. Second, additional adjustment for baseline CESD-10 scores did not materially change the results, with both G2 and G3 showing slower decline and frailty progression compared with G1. Third, excluding participants with baseline cognitive impairment or frailty yielded similar results, with stronger associations observed in G3 than G2. Fourth, excluding the stock-investment item from the definition of online social activity yielded a similar three-group trajectory solution, and the associations with cognition and frailty remained materially unchanged. Fifth, using a restrictive coding of ADL, IADL, and mobility deficits yielded similar results, with both G2 and G3 remaining associated with slower frailty progression compared with G1. Overall, findings were robust across sensitivity analyses (Supplementary Figure S3A–H; Supplementary Tables SA11–SA18).

## Discussion

This seven-year longitudinal study identified three joint trajectories of online and offline social activity engagement among middle-aged and older Chinese adults: the Persistently Low Social Activity Engagement Group, the Offline Social Activity Dominant Group, and the Online Social Activity Increasing Group. Compared with the Persistently Low Social Activity Engagement Group, both the Offline Social Activity Dominant Group and the Online Social Activity Increasing Group were associated with slower cognitive decline and frailty progression, with stronger associations observed in the Online Social Activity Increasing Group. Domain-specific analyses indicated that these associations were most evident for memory function, while weaker or non-significant associations were observed for orientation and executive function. These findings were generally robust across sensitivity analyses.

The association between offline activity engagement and slower cognitive decline is consistent with previous evidence suggesting that participation in socially, cognitively, or physically stimulating activities may support cognitive health in later life [30–34]. Offline social activities may support cognition through social interaction, physical movement, and cognitive stimulation, consistent with the “use it or lose it” hypothesis [12]. However, previous findings have not been entirely consistent. For example, studies using narrower definitions of social activity have reported weaker or null associations [35]. Our broader measure of offline activity may have captured more diverse forms of engagement, including social, recreational, voluntary, and educational participation. The finding that integrated online and offline social activity engagement was associated with even slower cognitive decline, particularly in memory function, extends previous research on activity engagement and cognitive aging. Participants in G3 showed the most favorable cognitive trajectories, which is consistent with prior evidence on combined activity engagement [28, 36] and studies linking internet use to better cognition [37–39]. Cognitive reserve may partly explain this association, as diverse online and offline activities may engage memory, executive control, and problem-solving processes [4, 40].

Our findings also suggest that activity engagement may be relevant to physical resilience. Both offline social activity dominant and online social activity increasing groups were associated with slower frailty progression, aligning with earlier studies linking activity participation to reduced frailty risk [5, 20, 38]. Offline social activities, such as sports, club participation, and volunteering, often involve physical and social components that may help maintain mobility, muscle strength, and social connectedness, thereby counteracting sarcopenia and functional decline, key mechanisms underlying frailty [41, 42]. Online social activities may mitigate frailty indirectly by supporting health information seeking, medication reminders, chronic disease self-monitoring, and communication with health or social support networks [37, 38, 43]. These complementary pathways may help explain the stronger associations observed in G3: online engagement may enhance self-regulation and health literacy, while offline engagement may preserve mobility and muscle function, which in turn may facilitate digital engagement over time [36]. Together, integrated activity engagement may contribute to both cognitive and physical resilience, underpinning the more favorable aging trajectories observed in the G3 group. However, these findings should be interpreted as longitudinal associations rather than causal effects.

Exploratory subgroup analyses showed some variation in point estimates, but formal three-way interaction tests and omnibus Wald tests provided no statistically significant evidence of effect modification by age, gender, residence, or *per capita* annual household income. Because some subgroup-specific estimates, particularly for G3, had wider confidence intervals, these findings should be interpreted cautiously and as hypothesis-generating rather than confirmatory. Unequal access to digital technologies and community resources may nevertheless limit opportunities for social and digital engagement among disadvantaged older adults, underscoring the importance of digital inclusion in healthy aging strategies.

These findings support multidimensional activity engagement as part of healthy aging strategies. First, in China, programs integrating accessible digital engagement with community-based social, cognitive, and physical activities may be particularly relevant. Attention to socioeconomic inequalities in digital access remains important. Second, as health and social services become increasingly digitalized, digital inclusion should be integrated into healthy aging policies, particularly for older adults with limited socioeconomic resources or restricted access to community activities. Third, because the Online Social Activity Increasing Group was relatively small and socioeconomically advantaged, future interventions should pay particular attention to digital inequality and avoid widening existing health disparities.

Several limitations should be acknowledged. First, activity engagement was self-reported and may be subject to recall or social desirability bias. Second, the online activity measure may not fully capture the diversity or intensity of digital engagement, and stock trading may partly reflect socioeconomic or financial characteristics. Findings were materially unchanged when online activity was defined using internet use alone, although residual exposure misclassification remains possible. Third, because social activity trajectories and changes in cognition and frailty were assessed over the same period, temporal precedence could not be established. Reverse causation and health selection therefore remain possible, even after excluding participants with baseline cognitive impairment or frailty. Accordingly, the findings should be interpreted as longitudinal associations rather than causal effects. Fourth, selective attrition may have affected the cognitive analytic sample, as excluded participants were generally older, less educated, and had poorer baseline cognition and higher frailty than included participants. In contrast, the risk of attrition bias appeared lower for frailty-related findings because included and excluded participants were largely comparable. Fifth, the Online Social Activity Increasing Group (G3) represented a relatively small proportion of the trajectory sample (5.2%; n = 749). Although classification diagnostics were satisfactory, estimates for this subgroup may be less precise than those for the larger groups, particularly in stratified analyses where further subdivision reduced sample sizes and statistical power to detect interaction effects. Therefore, findings concerning G3 should be interpreted cautiously and confirmed in larger samples. Finally, unmeasured factors such as personality, social networks, neighborhood environments, and genetic susceptibility may have influenced both activity engagement and aging outcomes.

In conclusion, this study identified distinct joint trajectories of online and offline social activity engagement among middle-aged and older adults in China. Offline-dominant and online social activity increasing trajectories were associated with slower cognitive decline and frailty progression, with the strongest associations observed in the Online Social Activity Increasing Group. Overall, these findings suggest that multidimensional activity engagement may be associated with healthier cognitive and physical aging trajectories among middle-aged and older adults. Given the relatively small and sociodemographically distinct G3 subgroup, larger studies with more detailed measures of digital engagement are needed to validate these findings.

## Data Availability

Publicly available datasets were analysed in this study. This data can be found here: https://charls.charlsdata.com. Access to the CHARLS dataset requires registration and approval from the CHARLS research team.
