# Supplementary material for: Joint trajectories of online and offline social activity, cognitive decline, and frailty progression among middle-aged and older adults in China
Source: Int J Public Health. 2026 Oct 14;71:1610116. doi: 10.3389/ijph.2026.1610116 (PMC13713438; doi:10.3389/ijph.2026.1610116)
Supplement: Supplementary file 1 [file DataSheet1.docx]

**Journal: International Journal of Public Health**

**Data sheet: Supplementary Appendix Material**

**Title: Joint Trajectories of Online and Offline Social Activity, Cognitive Decline, and Frailty Progression among Middle-aged and Older Adults in China**

**Supplementary Appendix information: Joint Trajectories of Online and Offline Social Activity, Cognitive Decline, and Frailty Progression among Middle-aged and Older Adults in China (China, 2011–2018).**

**Content**

[Supplementary Appendix Figure S1 | Participant flow diagram (China, 2011–2018).	3](#_Toc239548978)

[Supplementary Appendix Figure S2 | Heterogeneity analysis of longitudinal associations between joint online–offline social activity trajectory groups and changes in cognition and frailty across subgroups (China, 2011–2018).	4](#_Toc239548979)

[Supplementary Appendix Figure S3 | Sensitivity analyses of trajectories of cognitive decline and frailty progression by joint online–offline social activity trajectory groups (China, 2011–2018).	6](#_Toc239548980)

[Supplementary Appendix Table S1 | Baseline characteristics of participants included in and excluded from the cognitive function and frailty analytic samples (China, 2011–2018).	8](#_Toc239548981)

[Supplementary Appendix Table S2 | Model fit for joint online–offline social activity trajectory groups (China, 2011–2018).	9](#_Toc239548982)

[Supplementary Appendix Table S3 | Group trajectory model parameter estimates (based on the selected model: 112-122) (China, 2011–2018).	10](#_Toc239548983)

[Supplementary Appendix Table S4 | Classification diagnostics and posterior probabilities for joint online–offline social activity trajectory groups (China, 2011–2018).	11](#_Toc239548984)

[Supplementary Appendix Table S5 | List of 34 variables included in the physical frailty index (China, 2011–2018).	12](#_Toc239548985)

[Supplementary Appendix Table S6 | Longitudinal associations of joint online–offline social activity trajectory groups with changes in global cognition and frailty, full model results (China, 2011–2018).	14](#_Toc239548986)

[Supplementary Appendix Table S7 | Longitudinal associations of joint online–offline social activity trajectory groups with changes in cognitive subdomains, full model results (China, 2011–2018).	16](#_Toc239548987)

[Supplementary Appendix Table S8 | Three-way interaction analyses of heterogeneity in longitudinal associations with cognition and frailty (China, 2011–2018).	18](#_Toc239548988)

[Supplementary Appendix Table S9 | Heterogeneity analyses of mixed-effects models for cognitive decline across subgroups (China, 2011–2018).	20](#_Toc239548989)

[Supplementary Appendix Table S10 | Heterogeneity analyses of mixed-effects models for frailty progression across subgroups (China, 2011–2018).	21](#_Toc239548990)

[Supplementary Appendix Table S11 | Sensitivity analysis based on raw (unstandardized) cognition and frailty scores (China, 2011–2018).	22](#_Toc239548991)

[Supplementary Appendix Table S12 | Sensitivity analysis additionally adjusted for baseline depressive symptoms (China, 2011–2018).	24](#_Toc239548992)

[Supplementary Appendix Table S13 | Sensitivity analysis excluding individuals with baseline cognitive impairment or frailty (China, 2011–2018).	26](#_Toc239548993)

[Supplementary Appendix Table S14 | Sensitivity analysis of model fit for joint online–offline social activity trajectory groups (China, 2011–2018).	28](#_Toc239548994)

[Supplementary Appendix Table S15 | Sensitivity analysis of group trajectory model parameter estimates (based on the selected model: 112-122) (China, 2011–2018).	29](#_Toc239548995)

[Supplementary Appendix Table S16 | Sensitivity analysis of classification diagnostics and posterior probabilities for joint online–offline social activity trajectory groups after excluding the stock-related online activity item (China, 2011–2018).	30](#_Toc239548996)

[Supplementary Appendix Table S17 | Sensitivity analysis excluding stock-related online social activity from the trajectory definition (China, 2011–2018).	31](#_Toc239548997)

[Supplementary Appendix Table S18 | Sensitivity analysis using a restrictive coding of ADL, IADL, and mobility deficits in the frailty index (China, 2011–2018).	33](#_Toc239548998)

**Supplementary Appendix Figure S1 | Participant flow diagram (China, 2011–2018).**

Orientation function analytic sample: n = 12,926

777 participants were excluded due to age <45 years or missing age

2,591 participants were excluded due to missing baseline social activity data or lack of at least one follow-up activity assessment

14,340 participants were included in the joint trajectory analysis of online and offline social activities

Executive function analytic sample: n = 11,930

Memory function analytic sample: n = 12,070

1,414 participants were excluded because they had diagnosed memory-related diseases, missing baseline, or no follow-up orientation function data

2,270 participants were excluded because they had diagnosed memory-related diseases, missing baseline, or no follow-up memory function data

Frailty analytic sample: n = 13,960

380 participants were excluded because they had diagnosed memory-related diseases, missing baseline, or no follow-up frailty data

2,410 participants were excluded because they had diagnosed memory-related diseases, missing baseline, or no follow-up executive function data

4,017 participants were excluded because they had diagnosed memory-related diseases, missing baseline data, or no follow-up cognition data

16,931 participants retained

Sample size varied by outcome (total cognition, frailty, memory function, orientation function, and executive function)

Cognition analytic sample: n = 10,323

Baseline participants in Wave 1 (2011), N = 17,708

**Supplementary Appendix Figure S2 | Heterogeneity analysis of longitudinal associations between joint online–offline social activity trajectory groups and changes in cognition and frailty across subgroups (China, 2011–2018).**

**Figure S2(A) | Cognition: (G2 × Time) vs. (G1 × Time)**

**Figure S2(B) | Cognition: (G3 × Time) vs. (G1 × Time)**

**Figure S2(C) | Frailty: (G2 × Time) vs. (G1 × Time)**

**Figure S2(D) | Frailty: (G3 × Time) vs. (G1 × Time)**

**Abbreviations:** G1: Persistently Low Social Activity Engagement Group; G2: Offline Social Activity Dominant Group; G3: Online Social Activity Increasing Group.

**Supplementary Appendix Figure S3 |** **Sensitivity analyses of trajectories of cognitive decline and frailty progression by joint online–offline social activity trajectory groups (China,** **2011–2018).**

**Figure S3(A) | Cognition: based on raw (unstandardized) cognition score**

**Figure S3(B) | Frailty: based on raw (unstandardized) frailty score**

**Figure S3(C) | Cognition: additionally adjusted for baseline depressive symptoms**

**Figure S3(D) | Frailty: additionally adjusted for baseline depressive symptoms**

**Figure S3(E) | Cognition: excluding individuals with baseline cognitive impairment**

**Figure S3(F) | Frailty: excluding individuals with baseline frailty**

**Figure S3(G) | Cognition: excluding stock-related online social activity from the trajectory definition**

**Figure S3(H) | Frailty: excluding stock-related online social activity from the trajectory definition**

**Supplementary Appendix Table S1 | Baseline characteristics of participants included in and excluded from the cognitive function and frailty analytic samples (China, 2011–2018).**

| **Variables** | **Excluded from cognition sample, n=4,017** | **Included in cognition sample, n=10,323** | **p** | **Excluded from frailty sample, n=380** | **Included in frailty sample, n=13,960** | **p** |
| --- | --- | --- | --- | --- | --- | --- |
| Age, mean (SD), y | 61.69 (10.26) | 57.54 (8.55) | <0.001 | 61.81 (9.86) | 58.62 (9.22) | <0.001 |
| **Sex** |  |  | <0.001 |  |  | 0.110 |
| Male, n (%) | 1,452 (36.1%) | 5,358 (51.9%) |  | 196 (51.6%) | 6,614 (47.4%) |  |
| Female, n (%) | 2,565 (63.9%) | 4,965 (48.1%) |  | 184 (48.4%) | 7,346 (52.6%) |  |
| **Education** |  |  | <0.001 |  |  | 0.089 |
| Less than high school, n (%) | 3,825 (95.2%) | 8,858 (85.8%) |  | 347 (91.3%) | 12,336 (88.4%) |  |
| High school or equivalent, n (%) | 152 (3.8%) | 1,256 (12.2%) |  | 25 (6.6%) | 1,383 (9.9%) |  |
| College and higher, n (%) | 40 (1.0%) | 209 (2.0%) |  | 8 (2.1%) | 241 (1.7%) |  |
| **Marital status** |  |  | <0.001 |  |  | 0.840 |
| Not married, n (%) | 726 (18.1%) | 1,001 (9.7%) |  | 47 (12.4%) | 1,680 (12.0%) |  |
| Married, n (%) | 3,291 (81.9%) | 9,322 (90.3%) |  | 333 (87.6%) | 12,280 (88.0%) |  |
| **Residence** |  |  | <0.001 |  |  | 0.580 |
| Urban, n (%) | 1,196 (29.8%) | 4,343 (42.1%) |  | 152 (40.0%) | 5,387 (38.6%) |  |
| Rural, n (%) | 2,821 (70.2%) | 5,980 (57.9%) |  | 228 (60.0%) | 8,573 (61.4%) |  |
| **Baseline cognition score** | 10.70 (4.90) | 14.59 (4.33) | <0.001 | 14.07 (4.70) | 14.29 (4.49) | 0.450 |
| **Baseline frailty index** | 17.47 (14.32) | 11.62 (10.33) | <0.001 | 27.03 (17.97) | 13.04 (11.63) | <0.001 |

**Note:** Values are presented as mean (SD) for continuous variables and n (%) for categorical variables. Participants were classified as included in or excluded from the cognition and frailty analytic samples based on the prespecified eligibility criteria for each outcome. **Abbreviation:** SD, standard deviation.

**Supplementary Appendix Table S2 | Model fit for joint online–offline social activity trajectory groups (China, 2011–2018).**

| **Number of classes** | **Parameters of trajectory shape** | **Proportions per class** | **AvePP range** | **BIC** | **AIC** | **Entropy** |
| --- | --- | --- | --- | --- | --- | --- |
| 2 | 22-22 | 39.82%-60.18% | 0.87-0.91 | -68,904.47 | −68,851.47 | 0.638 |
| 3 | 222-222 | 6.62%-62.60% | 0.84-0.94 | -67,564.54 | −67,485.04 | 0.723 |
| 4 | 2222-2222 | 4.85%-47.90% | 0.75-0.92 | -67,030.87 | −66,924.87 | 0.625 |
| Selected model |  |  |  |  |  |  |
| 3 | 112-122 | 5.65%-47.49% | 0.86-0.92 | -67,397.18 | −67,329.04 | 0.719 |

**Abbreviations:** Parameters of trajectory shape: Online–offline social activities; Trajectory shape: 1 = linear, 2 = quadratic; AvePP, average posterior probability; BIC, Bayesian information criterion; AIC, Akaike information criterion.

We selected the models based on the criteria proposed by Lennon et al.^[1]^ and Hu et al.^[2]^ , considering statistical fit, classification quality, group size, and substantive interpretability. The criteria included: (1) comparative BIC and AIC values across candidate models;  (2) average posterior probabilities greater than 0.70 across all classes; (3) odds of correct classification based on posterior probabilities of group membership, ensuring they exceeded 5.0; (4) relative entropy values greater than 0.5; (5) substantively interpretable trajectory patterns; (6) adequate numbers of participants per group; and (7) close correspondence between the estimated group probabilities and the proportion of participants classified into those groups using the maximum posterior probability assignment rule. The final model was selected based on overall model adequacy, classification quality, parsimony, and substantive interpretability rather than the information criteria alone.

**Supplementary Appendix Table S3 | Group trajectory model parameter estimates (based on the selected model: 112-122) (China, 2011–2018).**

| **Online–offline social activities** | **Group** | **Parameter** | **Estimate** | **Standard error** | **p** | **95% CI** |
| --- | --- | --- | --- | --- | --- | --- |
| Online social activities | G1 | Intercept | -6.456 | 0.267 | <0.001 | [-6.980, -5.932] |
|  |  | Linear | 0.419 | 0.042 | <0.001 | [0.336, 0.502] |
|  | G2 | Intercept | -6.742 | 0.400 | <0.001 | [-7.527, -5.957] |
|  |  | Linear | 0.585 | 0.058 | <0.001 | [0.471, 0.699] |
|  | G3 | Intercept | -0.256 | 0.083 | 0.002 | [-0.419, -0.093] |
|  |  | Linear | 0.386 | 0.058 | <0.001 | [0.271, 0.500] |
|  |  | Quadratic | -0.028 | 0.008 | 0.001 | [-0.044, -0.011] |
| Offline social activities | G1 | Intercept | -0.701 | 0.027 | <0.001 | [-0.754, -0.649] |
|  |  | Linear | -0.062 | 0.006 | <0.001 | [-0.073, -0.050] |
|  | G2 | Intercept | 0.717 | 0.020 | <0.001 | [0.677, 0.757] |
|  |  | Linear | 0.134 | 0.012 | <0.001 | [0.112, 0.157] |
|  |  | Quadratic | -0.021 | 0.002 | <0.001 | [-0.024, -0.018] |
|  | G3 | Intercept | 1.190 | 0.047 | <0.001 | [1.098, 1.282] |
|  |  | Linear | 0.220 | 0.033 | <0.001 | [0.156, 0.285] |
|  |  | Quadratic | -0.032 | 0.005 | <0.001 | [-0.041, -0.023] |

**Abbreviations:** CI, confidence interval; G1, Persistently Low Social Activity Engagement Group; G2, Offline Social Activity Dominant Group; G3, Online Social Activity Increasing Group.

**Supplementary Appendix Table S4 | Classification diagnostics and posterior probabilities for joint online–offline social activity trajectory groups (China, 2011–2018).**

| **Trajectory**  **group** | **AvePP, mean (SD)** | **Range** | **OCC** | **OCCw** | **Estimated group probability, based on the sums of posterior probabilities (%)** | **Proportion assigned to group according to the maximum posterior probability assignment rule (%)** |
| --- | --- | --- | --- | --- | --- | --- |
| G1 | 0.869 (0.143) | 0.426–0.989 | 7.26 | 7.34 | 47.49% | 47.76% |
| G2 | 0.858 (0.157) | 0.442–0.995 | 6.80 | 6.84 | 46.86% | 47.02% |
| G3 | 0.922 (0.136) | 0.417–1.000 | 213.34 | 196.45 | 5.65% | 5.22% |

**Abbreviations:** AvePP, average posterior probability; SD, standard deviation; OCC, odds of correct classification; OCCw, odds of correct classification calculated using the estimated group probability; G1, Persistently Low Social Activity Engagement Group; G2, Offline Social Activity Dominant Group; G3, Online Social Activity Increasing Group.

**Supplementary Appendix Table S5 | List of 34 variables included in the physical frailty index (China, 2011–2018).**

| No. | Type of deficit | Description of the items | Cut-off value |
| --- | --- | --- | --- |
| 1 | Chronic disease | Self-reported diagnosed hypertension | Yes=1, no=0 |
| 2 |  | Self-reported diagnosed dyslipidemia | Yes=1, no=0 |
| 3 |  | Self-reported diagnosed diabetes or high blood sugar | Yes=1, no=0 |
| 4 |  | Self-reported diagnosed cancer or malignant tumor | Yes=1, no=0 |
| 5 |  | Self-reported diagnosed chronic lung diseases | Yes=1, no=0 |
| 6 |  | Self-reported diagnosed liver disease | Yes=1, no=0 |
| 7 |  | Self-reported diagnosed heart problems | Yes=1, no=0 |
| 8 |  | Self-reported diagnosed stroke | Yes=1, no=0 |
| 9 |  | Self-reported diagnosed kidney disease | Yes=1, no=0 |
| 10 |  | Self-reported diagnosed stomach or other digestive disease | Yes=1, no=0 |
| 11 |  | Self-reported diagnosed arthritis or rheumatism | Yes=1, no=0 |
| 12 |  | Self-reported diagnosed asthma | Yes=1, no=0 |
| 13 | sensory impairments | Self-reported hearing problem | Yes=1, no=0 |
| 14 |  | Self-reported vision problem | Yes=1, no=0 |
| 15 | ADL | ADL: any difficulty with dressing | Any difficulty=1; no difficulty=0* |
| 16 |  | ADL: any difficulty with bathing or showering | Any difficulty=1; no difficulty=0* |
| 17 |  | ADL: any difficulty with eating | Any difficulty=1; no difficulty=0* |
| 18 |  | ADL: any difficulty with getting in/out of bed | Any difficulty=1; no difficulty=0* |
| 19 |  | ADL: any difficulty with using the toilet | Any difficulty=1; no difficulty=0* |
| 20 |  | ADL: any difficulty with controlling urination and defecation | Any difficulty=1; no difficulty=0* |
| 21 | IADL | IADL: any difficulty with doing household chores | Any difficulty=1; no difficulty=0* |
| 22 |  | IADL: any difficulty with preparing hot meals | Any difficulty=1; no difficulty=0* |
| 23 |  | IADL: any difficulty with shopping for groceries | Any difficulty=1; no difficulty=0* |
| 24 |  | IADL: any difficulty with managing money | Any difficulty=1; no difficulty=0* |
| 25 |  | IADL: any difficulty with taking medications | Any difficulty=1; no difficulty=0* |
| 26 | Mobility | Mobility: any difficulty in jogging about 1 km | Any difficulty=1; no difficulty=0* |
| 27 |  | Mobility: any difficulty in walking 100 metres | Any difficulty=1; no difficulty=0* |
| 28 |  | Mobility: any difficulty in getting up from a chair after sitting for long periods | Any difficulty=1; no difficulty=0* |
| 29 |  | Mobility: any difficulty in climbing several flights of stairs without resting | Any difficulty=1; no difficulty=0* |
| 30 |  | Mobility: any difficulty in lifting or carrying weights over 10 jin | Any difficulty=1; no difficulty=0* |
| 31 |  | Mobility: any difficulty in picking up a coin from table | Any difficulty=1; no difficulty=0* |
| 32 |  | Mobility: any difficulty in stooping kneeling or crouching | Any difficulty=1; no difficulty=0* |
| 33 |  | Mobility: any difficulty in reaching or extending arms above shoulder level | Any difficulty=1; no difficulty=0* |
| 34 | Health status | Self-reported health status | Poor=1, fair=0.75, good=0.5, very good=0.25, excellent=0 |

**Abbreviations:** ADL, Activities of Daily Living; IADL, Instrumental Activities of Daily Living. **For ADL, IADL, and mobility items, the original CHARLS response options were: (1) no difficulty, (2) difficulty but can still do it, (3) difficulty and need help, and (4) cannot do it. Consistent with the Harmonized CHARLS Version D coding convention, response 1 was coded as 0 and responses 2–4 were coded as 1 in the primary frailty index.

**Supplementary Appendix Table S6 | Longitudinal associations of** **joint online–offline social activity trajectory groups with changes in global cognition and frailty, full model results (China, 2011–2018).**

|  | **Cognitive decline** | | **Frailty progression** | |
| --- | --- | --- | --- | --- |
|  | Coef [95% CI] | p | Coef [95% CI] | p |
| Time, years | -0.055 [-0.061, -0.049] | <0.001 | 0.095 [0.091, 0.100] | <0.001 |
| **Joint online–offline social activity trajectory groups** | | | | |
| G1 | Reference |  | Reference |  |
| G2 | 0.036 [0.016, 0.057] | 0.001 | -0.018 [-0.033, -0.003] | 0.016 |
| G3 | 0.100 [0.055, 0.145] | <0.001 | -0.020 [-0.055, 0.016] | 0.278 |
| **Joint online–offline social activity trajectory groups × time** | | | | |
| G1 × Time | Reference |  | Reference |  |
| G2 × Time | 0.010 [0.002, 0.018] | 0.011 | -0.014 [-0.020, -0.007] | <0.001 |
| G3 × Time | 0.044 [0.029, 0.060] | <0.001 | -0.041 [-0.055, -0.026] | <0.001 |
| Age | -0.008 [-0.009, -0.007] | <0.001 | 0.004 [0.003, 0.004] | <0.001 |
| Gender (male as reference) | | | | |
| Female | -0.076 [-0.097, -0.054] | <0.001 | 0.034 [0.017, 0.050] | <0.001 |
| Education level (less than high school as reference) | | | | |
| High school or equivalent | 0.127 [0.099, 0.155] | <0.001 | 0.000 [-0.023, 0.023] | 0.988 |
| College and higher | 0.144 [0.081, 0.207] | <0.001 | -0.023 [-0.074, 0.029] | 0.389 |
| Marital status (not married as reference) | | | | |
| Married | 0.058 [0.029, 0.088] | <0.001 | -0.022 [-0.042, -0.001] | 0.039 |
| Residence (urban as reference) | | | | |
| Rural | -0.085 [-0.104, -0.066] | <0.001 | 0.012 [-0.002, 0.027] | 0.088 |
| Per capita annual household income (below the median as reference) | | | | |
| At or above the median | 0.036 [0.018, 0.054] | <0.001 | -0.016 [-0.030, -0.003] | 0.017 |
| Medical insurance coverage (uninsured as reference) | | | | |
| Insured | 0.009 [-0.027, 0.044] | 0.633 | -0.010 [-0.036, 0.015] | 0.434 |
| Current employment status (not currently working as reference) | | | | |
| Currently working | -0.014 [-0.034, 0.007] | 0.185 | -0.055 [-0.070, -0.040] | <0.001 |
| Physical exercise (no as reference) | | | | |
| Yes | 0.018 [-0.001, 0.036] | 0.066 | -0.016 [-0.030, -0.001] | 0.031 |
| Alcohol consumption (no as reference) | | | | |
| Yes | 0.002 [-0.022, 0.026] | 0.860 | -0.028 [-0.047, -0.010] | 0.003 |
| Smoking (no as reference) | | | | |
| Yes | -0.026 [-0.048, -0.004] | 0.020 | -0.010 [-0.026,0.007] | 0.254 |
| Baseline cognition score (z-score) | 0.740 [0.731, 0.749] | <0.001 | — | — |
| Baseline frailty index (z-score) | — | — | 0.862 [0.855,0.869] | <0.001 |

**Note:** Models were adjusted for baseline age, gender, educational level, marital status, residence, per capita annual household income, medical insurance coverage, current employment status, physical exercise, alcohol consumption, smoking status, and baseline levels of the corresponding outcomes (z-scores). **Abbreviations:** Coef, coefficient; CI, confidence interval; G1, Persistently Low Social Activity Engagement Group; G2, Offline Social Activity Dominant Group; G3, Online Social Activity Increasing Group.

**Supplementary Appendix Table S7 | Longitudinal associations of joint online–offline social activity trajectory groups with changes in cognitive subdomains, full model results (China, 2011–2018).**

|  | **Memory function** | | **Orientation function** | | **Executive function** | |
| --- | --- | --- | --- | --- | --- | --- |
|  | Coef [95% CI] | p | Coef [95% CI] | p | Coef [95% CI] | p |
| Time, years | -0.050 [-0.056, -0.044] | <0.001 | -0.034 [-0.039, -0.029] | <0.001 | -0.035 [-0.040, -0.029] | <0.001 |
| **Joint online–offline social activity trajectory groups** | | | | | | |
| G1 | Reference |  | Reference |  | Reference |  |
| G2 | 0.034 [0.013, 0.055] | 0.002 | 0.046 [0.025, 0.066] | <0.001 | 0.038 [0.019, 0.057] | <0.001 |
| G3 | 0.096 [0.047, 0.144] | <0.001 | 0.083 [0.035, 0.130] | 0.001 | 0.086 [0.044, 0.129] | <0.001 |
| **Joint online–offline social activity trajectory groups × time** | | | | | | |
| G1 × Time | Reference |  | Reference |  | Reference |  |
| G2 × Time | 0.022 [0.014, 0.030] | <0.001 | 0.004 [-0.003, 0.011] | 0.243 | -0.001 [-0.008, 0.007] | 0.882 |
| G3 × Time | 0.073 [0.055, 0.090] | <0.001 | 0.011 [-0.004, 0.027] | 0.145 | 0.016 [0.001, 0.031] | 0.032 |
| Age | -0.012 [-0.013, -0.011] | <0.001 | -0.007 [-0.008, -0.006] | <0.001 | -0.004 [-0.005, -0.003] | <0.001 |
| Gender (male as reference) | | | | | | |
| Female | -0.028 [-0.050, -0.006] | 0.014 | -0.133 [-0.154, -0.112] | <0.001 | -0.114 [-0.134, -0.094] | <0.001 |
| Education level (less than high school as reference) | | | | | | |
| High school or equivalent | 0.172 [0.143, 0.202] | <0.001 | 0.102 [0.074, 0.131] | <0.001 | 0.080 [0.054, 0.106] | <0.001 |
| College and higher | 0.190 [0.122, 0.258] | <0.001 | 0.123 [0.058, 0.188] | <0.001 | 0.090 [0.031, 0.149] | 0.003 |
| Marital status (not married as reference) | | | | | | |
| Married | 0.064 [0.035, 0.093] | <0.001 | 0.034 [0.007, 0.062] | 0.014 | 0.043 [0.017, 0.070] | 0.001 |
| Residence (urban as reference) | | | | | | |
| Rural | -0.081 [-0.100, -0.062] | <0.001 | -0.123 [-0.141, -0.104] | <0.001 | -0.036 [-0.053, -0.019] | <0.001 |
| Per capita annual household income (below the median as reference) | | | | | | |
| At or above the median | 0.038 [0.020, 0.056] | <0.001 | 0.072 [0.055, 0.089] | <0.001 | 0.009 [-0.008, 0.025] | 0.295 |
| Medical insurance coverage (uninsured as reference) | | | | | | |
| Insured | 0.011 [-0.025, 0.046] | 0.552 | 0.017 [-0.017, 0.050] | 0.335 | 0.027 [-0.005, 0.059] | 0.101 |
| Current employment status (not currently working as reference) | | | | | | |
| Currently working | -0.011 [-0.031, 0.009] | 0.292 | -0.026 [-0.045, -0.007] | 0.008 | -0.012 [-0.030, 0.006] | 0.204 |
| Physical exercise (no as reference) | | | | | | |
| Yes | 0.019 [0.000, 0.038] | 0.047 | 0.004 [-0.015, 0.022] | 0.693 | -0.002 [-0.019, 0.015] | 0.839 |
| Alcohol consumption (no as reference) | | | | | | |
| Yes | -0.007 [-0.032, 0.017] | 0.557 | -0.012 [-0.036, 0.011] | 0.312 | 0.013 [-0.009, 0.035] | 0.238 |
| Smoking (no as reference) | | | | | | |
| Yes | -0.017 [-0.040, 0.005] | 0.128 | -0.041 [-0.062, -0.019] | <0.001 | -0.013 [-0.033, 0.007] | 0.195 |
| Baseline memory function (z-score) | 0.668 [0.659, 0.677] | <0.001 | — | — | — | — |
| Baseline orientation function (z-score) | — | — | 0.655 [0.646, 0.664] | <0.001 | — | — |
| Baseline executive function (z-score) | — | — | — | — | 0.710 [0.701, 0.718] | <0.001 |

**Note:** Models were adjusted for baseline age, gender, educational level, marital status, residence, per capita annual household income, medical insurance coverage, current employment status, physical exercise, alcohol consumption, smoking status, and baseline levels of the corresponding outcomes (z-score). **Abbreviations:** Coef, coefficient; CI, confidence interval; G1, Persistently Low Social Activity Engagement Group; G2, Offline Social Activity Dominant Group; G3, Online Social Activity Increasing Group.

**Supplementary Appendix Table S8 | Three-way interaction analyses of heterogeneity in longitudinal associations with cognition and frailty (China, 2011–2018).**

| **Heterogeneity variables and interactions** | **Cognitive decline** | | | **Frailty progression** | | |
| --- | --- | --- | --- | --- | --- | --- |
|  | Coef [95% CI] | p | Omnibus p for interaction | Coef [95% CI] | p | Omnibus p for interaction |
| **Gender (Ref. = Male)** |  |  | 0.841 |  |  | 0.738 |
| Female | -0.094 [-0.127, -0.061] | <0.001 |  | 0.029 [0.006, 0.052] | 0.013 |  |
| Female × time | 0.006 [-0.005, 0.017] | 0.297 |  | 0.011 [0.002, 0.020] | 0.021 |  |
| G2 × Female | 0.004 [-0.037, 0.044] | 0.863 |  | -0.010 [-0.039, 0.019] | 0.505 |  |
| G3 × Female | 0.081 [-0.003, 0.165] | 0.058 |  | -0.033 [-0.098, 0.033] | 0.330 |  |
| G2 × Female × time | 0.004 [-0.012, 0.019] | 0.638 |  | 0.001 [-0.012, 0.014] | 0.877 |  |
| G3 × Female × time | 0.008 [-0.024, 0.039] | 0.642 |  | -0.011 [-0.040, 0.018] | 0.477 |  |
| **Age (Ref. = <65 years)** |  |  | 0.562 |  |  | 0.489 |
| ≥ 65 years | -0.047 [-0.084, -0.011] | 0.011 |  | 0.016 [-0.007, 0.040] | 0.176 |  |
| ≥ 65 years × time | -0.062 [-0.075, -0.048] | <0.001 |  | 0.055 [0.045, 0.066] | <0.001 |  |
| G2 × ≥ 65 years | -0.008 [-0.057, 0.042] | 0.767 |  | -0.015 [-0.049, 0.018] | 0.376 |  |
| G3 × ≥65 years | 0.050 [-0.093, 0.194] | 0.493 |  | -0.018 [-0.132, 0.096] | 0.753 |  |
| G2 × ≥ 65 years × time | 0.010 [-0.009, 0.029] | 0.296 |  | -0.009 [-0.024, 0.006] | 0.252 |  |
| G3 × ≥ 65 years × time | -0.002 [-0.057, 0.054] | 0.957 |  | -0.013 [-0.063, 0.038] | 0.617 |  |
| **Residence (Ref. = Urban)** |  |  | 0.736 |  |  | 0.511 |
| Rural | -0.058 [-0.091, -0.026] | <0.001 |  | 0.009 [-0.014, 0.031] | 0.456 |  |
| Rural × time | -0.008 [-0.020, 0.004] | 0.187 |  | -0.001 [-0.010, 0.009] | 0.891 |  |
| G2 × Rural | -0.018 [-0.061, 0.024] | 0.392 |  | 0.003 [-0.027, 0.034] | 0.826 |  |
| G3 × Rural | -0.018 [-0.131, 0.094] | 0.750 |  | 0.017 [-0.070, 0.105] | 0.694 |  |
| G2 × Rural × time | -0.006 [-0.022, 0.010] | 0.442 |  | 0.005 [-0.008, 0.019] | 0.454 |  |
| G3 × Rural × time | 0.000 [-0.042, 0.041] | 0.983 |  | -0.014 [-0.051, 0.024] | 0.467 |  |
| **Per capita annual household income (Ref. = below the median)** | | | 0.815 |  |  | 0.763 |
| At or above the median | 0.008 [-0.023, 0.038] | 0.614 |  | -0.007 [-0.028, 0.015] | 0.540 |  |
| At or above the median × time | 0.010 [-0.001, 0.021] | 0.089 |  | -0.015 [-0.024, -0.006] | 0.001 |  |
| G2 × At or above the median | 0.029 [-0.012, 0.070] | 0.164 |  | 0.005 [-0.024, 0.035] | 0.713 |  |
| G3 × At or above the median | 0.013 [-0.113, 0.140] | 0.836 |  | 0.030 [-0.071, 0.131] | 0.557 |  |
| G2 × At or above the median × time | -0.001 [-0.016, 0.015] | 0.910 |  | 0.005 [-0.008, 0.018] | 0.462 |  |
| G3 × At or above the median × time | -0.015 [-0.062, 0.031] | 0.522 |  | 0.003 [-0.041, 0.046] | 0.901 |  |

**Note:** Separate mixed-effects models were fitted for gender, age, residence, and per capita annual household income. Each model included a three-way interaction between trajectory group, time, and the corresponding heterogeneity variable. The omnibus P value for interaction was obtained from a joint Wald test of the G2 × time × heterogeneity-variable and G3 × time × heterogeneity-variable terms. Models were adjusted for baseline age, gender, educational level, marital status, residence, per capita annual household income, medical insurance coverage, current employment status, physical exercise, alcohol consumption, smoking status, and the baseline value of the corresponding outcomes (z-score), except that the heterogeneity variable was omitted from its corresponding model. **Abbreviations:** Coef, coefficient; CI, confidence interval; G1, Persistently Low Social Activity Engagement Group; G2, Offline Social Activity Dominant Group; G3, Online Social Activity Increasing Group.

**Supplementary Appendix Table S9 | Heterogeneity analyses of mixed-effects models for cognitive decline across subgroups (China, 2011–2018).**

| **Variables** | **Age <65 years** | | **Age ≥65 years** | | **Male** | | **Female** | |
| --- | --- | --- | --- | --- | --- | --- | --- | --- |
|  | Coef [95% CI] | p | Coef [95% CI] | p | Coef [95% CI] | p | Coef [95% CI] | p |
| **Panel A. Age and gender subgroups** | | | | | | | | |
| Time, years | -0.042 [-0.048, -0.036] | <0.001 | -0.104 [-0.117, -0.091] | <0.001 | -0.058 [-0.065, -0.050] | <0.001 | -0.052 [-0.060, -0.043] | <0.001 |
| **Joint online–offline social activity trajectory groups** | | | | | | | | |
| G2 | 0.043 [0.020, 0.066] | <0.001 | 0.025 [-0.022, 0.073] | 0.293 | 0.045 [0.018, 0.073] | 0.001 | 0.031 [0.001, 0.062] | 0.046 |
| G3 | 0.132 [0.085, 0.179] | <0.001 | 0.123 [-0.029, 0.274] | 0.112 | 0.105 [0.047, 0.162] | <0.001 | 0.088 [0.017, 0.160] | 0.015 |
| **Joint online–offline social activity trajectory groups × time** | | | | | | | | |
| G2 × Time | 0.007 [-0.002, 0.015] | 0.123 | 0.017 [-0.002, 0.035] | 0.074 | 0.008 [-0.002, 0.018] | 0.128 | 0.012 [0.000, 0.023] | 0.043 |
| G3 × Time | 0.037 [0.020, 0.053] | <0.001 | 0.036 [-0.021, 0.093] | 0.217 | 0.041 [0.021, 0.062] | <0.001 | 0.049 [0.024, 0.073] | <0.001 |
| Sample size, n | 8,089 | | 2,111 | | 5,300 | | 4,900 | |
| **Panel B. Residence and per capita annual household income subgroups** | | | | | | | | |
| Time, years | -0.050 [-0.059, -0.040] | <0.001 | -0.058 [-0.065, -0.050] | <0.001 | -0.060 [-0.068, -0.051] | <0.001 | -0.050 [-0.058, -0.042] | <0.001 |
| **Joint online–offline social activity trajectory groups** | | | | | | | | |
| G2 | 0.046 [0.014, 0.077] | 0.005 | 0.034 [0.006, 0.061] | 0.016 | 0.023 [-0.008, 0.055] | 0.148 | 0.051 [0.024, 0.078] | <0.001 |
| G3 | 0.114 [0.064, 0.164] | <0.001 | 0.090 [-0.017, 0.198] | 0.100 | 0.096 [-0.032, 0.224] | 0.141 | 0.113 [0.066, 0.161] | <0.001 |
| **Joint online–offline social activity trajectory groups × time** | | | | | | | | |
| G2 × Time | 0.013 [0.001, 0.025] | 0.034 | 0.007 [-0.003, 0.017] | 0.185 | 0.010 [-0.002, 0.021] | 0.110 | 0.009 [-0.002, 0.019] | 0.098 |
| G3 × Time | 0.040 [0.023, 0.058] | <0.001 | 0.040 [0.002, 0.078] | 0.040 | 0.054 [0.008, 0.099] | 0.021 | 0.039 [0.022, 0.055] | <0.001 |
| Sample size, n | 4,281 | | 5,919 | | 4,595 | | 5,605 | |

**Note:** Models were adjusted for baseline age, gender, educational level, marital status, residence, per capita annual household income, medical insurance coverage, current employment status, physical exercise, alcohol consumption, smoking status, and baseline cognition (z-score), except that the stratification variable was omitted from the corresponding subgroup-specific model. Sample size represents the number of unique participants included in the estimation sample of the corresponding subgroup-specific mixed-effects model. **Abbreviations:** Coef, coefficient; CI, confidence interval; G1, Persistently Low Social Activity Engagement Group; G2, Offline Social Activity Dominant Group; G3, Online Social Activity Increasing Group.

**Supplementary Appendix Table S10 | Heterogeneity analyses of mixed-effects models for frailty progression across subgroups (China, 2011–2018).**

| **Variables** | **Age <65 years** | | **Age ≥65 years** | | **Male** | | **Female** | |
| --- | --- | --- | --- | --- | --- | --- | --- | --- |
|  | Coef [95% CI] | p | Coef [95% CI] | p | Coef [95% CI] | p | Coef [95% CI] | p |
| **Panel A. Age and gender subgroups** | | | | | | | | |
| Time, years | 0.081 [0.076, 0.085] | <0.001 | 0.136 [0.126, 0.147] | <0.001 | 0.090 [0.084, 0.097] | <0.001 | 0.100 [0.094, 0.106] | <0.001 |
| **Joint online–offline social activity trajectory groups** | | | | | | | | |
| G2 | -0.017 [-0.032, -0.001] | 0.036 | -0.035 [-0.071, 0.001] | 0.054 | -0.010 [-0.030, 0.010] | 0.318 | -0.025 [-0.046, -0.003] | 0.023 |
| G3 | -0.030 [-0.064, 0.005] | 0.093 | -0.068 [-0.208, 0.072] | 0.343 | -0.003 [-0.047, 0.041] | 0.907 | -0.044 [-0.101, 0.013] | 0.132 |
| **Joint online–offline social activity trajectory groups × time** | | | | | | | | |
| G2 × Time | -0.010 [-0.016, -0.003] | 0.004 | -0.019 [-0.035, -0.003] | 0.023 | -0.015 [-0.024, -0.006] | 0.001 | -0.013 [-0.022, -0.004] | 0.004 |
| G3 × Time | -0.030 [-0.043, -0.016] | <0.001 | -0.043 [-0.102, 0.017] | 0.160 | -0.036 [-0.054, -0.017] | <0.001 | -0.045 [-0.068, -0.023] | <0.001 |
| Sample size, n | 10,350 | | 3,391 | | 6,523 | | 7,218 | |
| **Panel B. Residence and per capita annual household income subgroups** | | | | | | | | |
| Time, years | 0.096 [0.088, 0.104] | <0.001 | 0.095 [0.089, 0.100] | <0.001 | 0.101 [0.095, 0.108] | <0.001 | 0.087 [0.081, 0.093] | <0.001 |
| **Joint online–offline social activity trajectory groups** | | | | | | | | |
| G2 | -0.019 [-0.041, 0.004] | 0.102 | -0.018 [-0.037, 0.002] | 0.071 | -0.026 [-0.048, -0.003] | 0.026 | -0.013 [-0.031, 0.006] | 0.179 |
| G3 | -0.026 [-0.064, 0.012] | 0.183 | -0.002 [-0.085, 0.082] | 0.971 | -0.061 [-0.168, 0.046] | 0.263 | -0.010 [-0.044, 0.025] | 0.574 |
| **Joint online–offline social activity trajectory groups × time** | | | | | | | | |
| G2 × Time | -0.017 [-0.027, -0.007] | <0.001 | -0.012 [-0.020, -0.004] | 0.005 | -0.015 [-0.024, -0.005] | 0.003 | -0.010 [-0.019, -0.002] | 0.016 |
| G3 × Time | -0.039 [-0.055, -0.023] | <0.001 | -0.052 [-0.087, -0.018] | 0.003 | -0.036 [-0.080, 0.008] | 0.111 | -0.034 [-0.048, -0.019] | <0.001 |
| Sample size, n | 5,285 | | 8,456 | | 6,846 | | 6,895 | |

**Note:** Models were adjusted for baseline age, gender, educational level, marital status, residence, per capita annual household income, medical insurance coverage, current employment status, physical exercise, alcohol consumption, smoking status, and baseline frailty (z-score), except that the stratification variable was omitted from the corresponding subgroup-specific model. Sample size represents the number of unique participants included in the estimation sample of the corresponding subgroup-specific mixed-effects model. **Abbreviations:** Coef, coefficient; CI, confidence interval; G1, Persistently Low Social Activity Engagement Group; G2, Offline Social Activity Dominant Group; G3, Online Social Activity Increasing Group.

**Supplementary Appendix Table S11 | Sensitivity analysis based on raw (unstandardized) cognition and frailty scores (China, 2011–2018).**

|  | **Cognitive decline** | | | **Frailty progression** | | |
| --- | --- | --- | --- | --- | --- | --- |
|  | Coef | 95% CI | p | Coef | 95% CI | p |
| Time, years | -0.171 | [-0.197, -0.144] | <0.001 | 1.107 | [1.055,1.160] | <0.001 |
| **Joint online–offline social activity trajectory groups** | | | | | | |
| G1 | Reference |  |  | Reference |  |  |
| G2 | 0.167 | [0.079,0.255] | <0.001 | -0.211 | [-0.382, -0.040] | 0.016 |
| G3 | 0.476 | [0.288,0.663] | <0.001 | -0.229 | [-0.642,0.184] | 0.278 |
| **Joint online–offline social activity trajectory groups × time** | | | | | | |
| G1 × Time | Reference |  |  | Reference |  |  |
| G2 × Time | 0.038 | [0.002,0.074] | 0.038 | -0.161 | [-0.235, -0.087] | <0.001 |
| G3 × Time | 0.199 | [0.129,0.270] | <0.001 | -0.472 | [-0.639, -0.304] | <0.001 |
| Age | -0.037 | [-0.042, -0.032] | <0.001 | 0.041 | [0.032,0.051] | <0.001 |
| Gender (male as reference) | | | | | | |
| Female | -0.206 | [-0.299, -0.114] | <0.001 | 0.392 | [0.202,0.582] | <0.001 |
| Education level (less than high school as reference) | | | | | | |
| High school or equivalent | 0.581 | [0.464,0.699] | <0.001 | 0.002 | [-0.262,0.266] | 0.988 |
| College and higher | 0.657 | [0.390,0.924] | <0.001 | -0.264 | [-0.865,0.336] | 0.389 |
| Marital status (not married as reference) | | | | | | |
| Married | 0.242 | [0.116,0.368] | <0.001 | -0.252 | [-0.491, -0.013] | 0.039 |
| Residence (urban as reference) | | | | | | |
| Rural | -0.326 | [-0.407, -0.245] | <0.001 | 0.144 | [-0.021,0.310] | 0.088 |
| Per capita annual household income (below the median as reference) | | | | | | |
| At or above the median | 0.157 | [0.080,0.234] | <0.001 | -0.192 | [-0.350, -0.034] | 0.017 |
| Medical insurance coverage (uninsured as reference) | | | | | | |
| Insured | 0.036 | [-0.115,0.187] | 0.637 | -0.119 | [-0.417,0.179] | 0.434 |
| Current employment status (not currently working as reference) | | | | | | |
| Currently working | -0.055 | [-0.141,0.032] | 0.215 | -0.641 | [-0.816, -0.466] | <0.001 |
| Physical exercise (no as reference) | | | | | | |
| Yes | 0.069 | [-0.011,0.148] | 0.093 | -0.181 | [-0.347, -0.016] | 0.031 |
| Alcohol consumption (no as reference) | | | | | | |
| Yes | 0.000 | [-0.101,0.101] | 0.994 | -0.331 | [-0.547, -0.116] | 0.003 |
| Smoking (no as reference) | | | | | |  |
| Yes | -0.076 | [-0.169,0.017] | 0.109 | -0.113 | [-0.308,0.082] | 0.254 |
| Baseline cognition score (raw score) | 0.734 | [0.725,0.744] | <0.001 | — | | |
| Baseline frailty index (raw score) | — | | | 0.862 | [0.855,0.869] | <0.001 |

**Note:** Models were adjusted for baseline age, gender, educational level, marital status, residence, per capita annual household income, medical insurance coverage, current employment status, physical exercise, alcohol consumption, smoking status, and baseline level of the corresponding outcomes (raw score). **Abbreviations:** Coef, coefficient; CI, confidence interval; G1, Persistently Low Social Activity Engagement Group; G2, Offline Social Activity Dominant Group; G3, Online Social Activity Increasing Group.

**Supplementary Appendix Table S12 | Sensitivity analysis additionally adjusted for baseline depressive symptoms (China, 2011–2018).**

|  | **Cognitive decline** | | | **Frailty progression** | | |
| --- | --- | --- | --- | --- | --- | --- |
|  | Coef | 95% CI | p | Coef | 95% CI | p |
| Time, years | -0.055 | [-0.061, -0.049] | <0.001 | 0.095 | [0.091,0.100] | <0.001 |
| **Joint online–offline social activity trajectory groups** | | | | | | |
| G1 | Reference |  |  | Reference |  |  |
| G2 | 0.033 | [0.013,0.054] | 0.002 | -0.015 | [-0.030, -0.001] | 0.041 |
| G3 | 0.095 | [0.050,0.140] | <0.001 | -0.016 | [-0.051,0.020] | 0.389 |
| **Joint online–offline social activity trajectory groups × time** | | | | | | |
| G1 × Time | Reference |  |  | Reference |  |  |
| G2 × Time | 0.010 | [0.002,0.018] | 0.011 | -0.014 | [-0.020, -0.007] | <0.001 |
| G3 × Time | 0.044 | [0.029,0.060] | <0.001 | -0.041 | [-0.055, -0.026] | <0.001 |
| Age | -0.008 | [-0.009, -0.007] | <0.001 | 0.004 | [0.003,0.005] | <0.001 |
| Gender (male as reference) | | | | | | |
| Female | -0.071 | [-0.093, -0.049] | <0.001 | 0.027 | [0.010,0.043] | 0.002 |
| Education level (less than high school as reference) | | | | | | |
| High school or equivalent | 0.124 | [0.096,0.152] | <0.001 | 0.006 | [-0.017,0.029] | 0.624 |
| College and higher | 0.140 | [0.077,0.204] | <0.001 | -0.017 | [-0.069,0.035] | 0.522 |
| Marital status (not married as reference) | | | | | | |
| Married | 0.052 | [0.023,0.082] | 0.001 | -0.013 | [-0.034,0.007] | 0.210 |
| Residence (urban as reference) | | | | | | |
| Rural | -0.081 | [-0.100, -0.062] | <0.001 | 0.007 | [-0.007,0.021] | 0.340 |
| Per capita annual household income (below the median as reference) | | | | | | |
| At or above the median | 0.031 | [0.013,0.049] | 0.001 | -0.012 | [-0.026,0.002] | 0.086 |
| Medical insurance coverage (uninsured as reference) | | | | | | |
| Insured | 0.009 | [-0.026,0.044] | 0.618 | -0.008 | [-0.034,0.018] | 0.552 |
| Current employment status (not currently working as reference) | | | | | | |
| Currently working | -0.016 | [-0.036,0.004] | 0.118 | -0.057 | [-0.072, -0.042] | <0.001 |
| Physical exercise (no as reference) | | | | | | |
| Yes | 0.017 | [-0.001,0.036] | 0.067 | -0.016 | [-0.030, -0.002] | 0.026 |
| Alcohol consumption (no as reference) | | | | | | |
| Yes | 0.001 | [-0.023,0.025] | 0.940 | -0.029 | [-0.047, -0.010] | 0.002 |
| Smoking (no as reference) | | | | | | |
| Yes | -0.025 | [-0.047, -0.003] | 0.025 | -0.012 | [-0.029,0.004] | 0.147 |
| CESD-10 score (raw score) | -0.004 | [-0.005, -0.002] | <0.001 | 0.005 | [0.004,0.006] | <0.001 |
| Baseline cognition score (z-score) | 0.735 | [0.726,0.745] | <0.001 | — | | |
| Baseline frailty index (z-score) | — | | | 0.847 | [0.840,0.855] | <0.001 |

**Note:** Models were adjusted for baseline age, gender, educational level, marital status, residence, per capita annual household income, medical insurance coverage, current employment status, physical exercise, alcohol consumption, smoking status, baseline CES-D-10 score (raw score), and baseline level of the corresponding outcomes (z-score). **Abbreviations:** Coef, coefficient; CI, confidence interval; CES-D-10, 10-item Center for Epidemiologic Studies Depression Scale; G1, Persistently Low Social Activity Engagement Group; G2, Offline Social Activity Dominant Group; G3, Online Social Activity Increasing Group.

**Supplementary Appendix Table S13 | Sensitivity analysis excluding individuals with baseline cognitive impairment or frailty (China, 2011–2018).**

|  | **Cognitive decline** | | | **Frailty progression** | | |
| --- | --- | --- | --- | --- | --- | --- |
|  | Coef | 95% CI | p | Coef | 95% CI | p |
| Time, years | -0.090 | [-0.098, -0.083] | <0.001 | 0.186 | [0.178,0.194] | <0.001 |
| **Joint online–offline social activity trajectory groups** | | | | | |  |
| G1 | Reference |  |  | Reference |  |  |
| G2 | 0.038 | [0.010,0.065] | 0.007 | -0.028 | [-0.053, -0.002] | 0.032 |
| G3 | 0.100 | [0.044,0.156] | <0.001 | -0.007 | [-0.066,0.051] | 0.802 |
| **Joint online–offline social activity trajectory groups × time** | | | | | |  |
| G1 × Time | Reference |  |  | Reference |  |  |
| G2 × Time | 0.017 | [0.007,0.027] | 0.001 | -0.027 | [-0.038, -0.015] | <0.001 |
| G3 × Time | 0.072 | [0.052,0.092] | <0.001 | -0.084 | [-0.108, -0.059] | <0.001 |
| Age | -0.009 | [-0.011, -0.008] | <0.001 | 0.006 | [0.004,0.007] | <0.001 |
| Gender (male as reference) | | | | | |  |
| Female | -0.088 | [-0.116, -0.059] | <0.001 | 0.059 | [0.030,0.087] | <0.001 |
| Education level (less than high school as reference) | | | | | |  |
| High school or equivalent | 0.155 | [0.121,0.190] | <0.001 | -0.004 | [-0.042,0.034] | 0.839 |
| College and higher | 0.188 | [0.110,0.266] | <0.001 | -0.047 | [-0.132,0.038] | 0.277 |
| Marital status (not married as reference) | | | | | |  |
| Married | 0.060 | [0.021,0.100] | 0.003 | -0.047 | [-0.084, -0.010] | 0.013 |
| Residence (urban as reference) | | | | | |  |
| Rural | -0.106 | [-0.131, -0.081] | <0.001 | 0.006 | [-0.019,0.030] | 0.648 |
| Per capita annual household income (below the median as reference) | | | | | |  |
| At or above the median | 0.054 | [0.030,0.078] | <0.001 | -0.040 | [-0.063, -0.016] | 0.001 |
| Medical insurance coverage (uninsured as reference) | | | | | |  |
| Insured | 0.000 | [-0.047,0.048] | 0.990 | -0.027 | [-0.071,0.018] | 0.236 |
| Current employment status (not currently working as reference) | | | | | |  |
| Currently working | -0.009 | [-0.036,0.018] | 0.509 | -0.094 | [-0.121, -0.068] | <0.001 |
| Physical exercise (no as reference) | | | | | |  |
| Yes | 0.026 | [0.002,0.051] | 0.036 | -0.011 | [-0.036,0.013] | 0.355 |
| Alcohol consumption (no as reference) | | | | | |  |
| Yes | 0.000 | [-0.031,0.031] | 0.991 | -0.046 | [-0.078, -0.015] | 0.004 |
| Smoking (no as reference) | | | | | |  |
| Yes | -0.035 | [-0.064, -0.007] | 0.014 | -0.013 | [-0.042,0.016] | 0.380 |
| Baseline cognition score (z-score) | 0.740 | [0.728,0.753] | <0.001 | — | | |
| Baseline frailty index (z-score) | — | | | 0.917 | [0.905,0.928] | <0.001 |

**Note:** For the cognition analysis, participants with baseline cognitive impairment, defined as a global cognition score more than 1 SD below the mean within 5-year age groups, were excluded. For the frailty analysis, participants with baseline frailty (frailty index ≥25) were excluded. Models were adjusted for baseline age, gender, educational level, marital status, residence, per capita annual household income, medical insurance coverage, current employment status, physical exercise, alcohol consumption, smoking status, and baseline level of the corresponding outcomes (z score). **Abbreviations:** Coef, coefficient; CI, confidence interval; G1, Persistently Low Social Activity Engagement Group; G2, Offline Social Activity Dominant Group; G3, Online Social Activity Increasing Group.

**Supplementary Appendix Table S14 | Sensitivity analysis of model fit for joint online–offline social activity trajectory groups (China, 2011–2018).**

| Number of classes | Parameters of trajectory shape | Proportions per class | AvePP | BIC | AIC | Entropy |
| --- | --- | --- | --- | --- | --- | --- |
| 2 | 22-22 | 40.15%-59.85% | 0.88-0.90 | -68,779.82 | -68,713.00 | 0.636 |
| 3 | 222-222 | 5.53%-47.74% | 0.86-0.92 | -67,334.86 | -67,234.64 | 0.718 |
| 4 | 2222-2222 | 7.06%-45.92% | 0.75-0.93 | -67,078.69 | -66,945.05 | 0.639 |
| Selected model |  |  |  |  |  |  |
| 3 | 112-122 | 5.63%-47.50% | 0.86-0.92 | -67,330.32 | -67,244.41 | 0.717 |

**Abbreviations:** Parameters of trajectory shape: Online–offline social activities; Trajectory shape: 1 = linear, 2 = quadratic; AvePP, average posterior probability; BIC, Bayesian information criterion; AIC, Akaike information criterion.

**Supplementary Appendix Table S15 | Sensitivity analysis of group trajectory model parameter estimates (based on the selected model: 112-122) (China, 2011–2018).**

| **Online–offline social activities** | **Group** | **Parameter** | **Estimate** | **Standard error** | **p** | **95% CI** |
| --- | --- | --- | --- | --- | --- | --- |
| Online social activities | G1 | Intercept | -6.426 | 0.263 | <0.001 | [-6.941, -5.910] |
|  |  | Linear | 0.416 | 0.042 | <0.001 | [0.334,0.498] |
|  | G2 | Intercept | -7.026 | 0.489 | <0.001 | [-7.984, -6.068] |
|  |  | Linear | 0.624 | 0.071 | <0.001 | [0.486, 0.763] |
|  | G3 | Intercept | -0.321 | 0.085 | <0.001 | [-0.487, -0.155] |
|  |  | Linear | 0.375 | 0.058 | <0.001 | [0.262, 0.489] |
|  |  | Quadratic | -0.026 | 0.008 | 0.002 | [-0.043, -0.010] |
| Offline social activities | G1 | Intercept | -0.702 | 0.027 | <0.001 | [-0.754, -0.649] |
|  |  | Linear | -0.061 | 0.006 | <0.001 | [-0.073, -0.050] |
|  | G2 | Intercept | 0.718 | 0.020 | <0.001 | [0.678, 0.758] |
|  |  | Linear | 0.133 | 0.012 | <0.001 | [0.111, 0.156] |
|  |  | Quadratic | -0.021 | 0.002 | <0.001 | [-0.024, -0.018] |
|  | G3 | Intercept | 1.190 | 0.047 | <0.001 | [1.098, 1.283] |
|  |  | Linear | 0.221 | 0.033 | <0.001 | [0.156, 0.286] |
|  |  | Quadratic | -0.032 | 0.005 | <0.001 | [-0.041, -0.023] |

**Abbreviations:** CI, confidence interval; G1, Persistently Low Social Activity Engagement Group; G2, Offline Social Activity Dominant Group; G3, Online Social Activity Increasing Group.

**Supplementary Appendix Table S16 | Sensitivity analysis of classification diagnostics and posterior probabilities for joint online–offline social activity trajectory groups after excluding the stock-related online activity item (China, 2011–2018).**

| **Trajectory**  **group** | **AvePP, mean (SD)** | **Range** | **OCC** | **OCCw** | **Estimated group probability, based on the sums of posterior probabilities (%)** | **Proportion assigned to group according to the maximum posterior probability assignment rule (%)** |
| --- | --- | --- | --- | --- | --- | --- |
| G1 | 0.869 (0.144) | 0.430–0.990 | 7.23 | 7.32 | 47.50% | 47.80% |
| G2 | 0.858 (0.157) | 0.398–0.995 | 6.81 | 6.84 | 46.87% | 46.98% |
| G3 | 0.917 (0.142) | 0.428–1.000 | 199.20 | 183.90 | 5.63% | 5.22% |

**Abbreviations:** AvePP, average posterior probability; SD, standard deviation; OCC, odds of correct classification; OCCw, odds of correct classification calculated using the estimated group probability; G1, Persistently Low Social Activity Engagement Group; G2, Offline Social Activity Dominant Group; G3, Online Social Activity Increasing Group.

**Supplementary Appendix Table S17 | Sensitivity analysis excluding stock-related online social activity from the trajectory definition (China, 2011–2018).**

|  | **Cognitive decline** | | | **Frailty progression** | | |
| --- | --- | --- | --- | --- | --- | --- |
|  | Coef | 95% CI | p | Coef | 95% CI | p |
| Time, years | -0.055 | [-0.061, -0.049] | <0.001 | 0.095 | [0.091,0.100] | <0.001 |
| **Joint online–offline social activity trajectory groups** | | | | | | |
| G1 | Reference |  |  | Reference |  |  |
| G2 | 0.036 | [0.015,0.057] | 0.001 | -0.018 | [-0.033, -0.003] | 0.017 |
| G3 | 0.100 | [0.055,0.145] | <0.001 | -0.018 | [-0.053,0.018] | 0.327 |
| **Joint online–offline social activity trajectory groups × time** | | | | | | |
| G1 × Time | Reference |  |  | Reference |  |  |
| G2 × Time | 0.010 | [0.002,0.018] | 0.010 | -0.014 | [-0.020, -0.008] | <0.001 |
| G3 × Time | 0.045 | [0.029,0.060] | <0.001 | -0.040 | [-0.054, -0.026] | <0.001 |
| Age | -0.008 | [-0.009, -0.007] | <0.001 | 0.004 | [0.003,0.004] | <0.001 |
| Gender (male as reference) | | | | | | |
| Female | -0.075 | [-0.097, -0.054] | <0.001 | 0.034 | [0.017,0.050] | <0.001 |
| Education level (less than high school as reference) | | | | | | |
| High school or equivalent | 0.127 | [0.099,0.155] | <0.001 | 0.000 | [-0.023,0.022] | 0.977 |
| College and higher | 0.146 | [0.082,0.209] | <0.001 | -0.024 | [-0.076,0.027] | 0.359 |
| Marital status (not married as reference) | | | | | | |
| Married | 0.058 | [0.029,0.088] | <0.001 | -0.022 | [-0.042, -0.001] | 0.039 |
| Residence (urban as reference) | | | | | | |
| Rural | -0.085 | [-0.104, -0.066] | <0.001 | 0.013 | [-0.002,0.027] | 0.083 |
| Per capita annual household income (below the median as reference) | | | | | | |
| At or above the median | 0.036 | [0.018,0.054] | <0.001 | -0.017 | [-0.030, -0.003] | 0.017 |
| Medical insurance coverage (uninsured as reference) | | | | | | |
| Insured | 0.008 | [-0.027,0.044] | 0.643 | -0.010 | [-0.036,0.015] | 0.434 |
| Current employment status (not currently working as reference) | | | | | | |
| Currently working | -0.014 | [-0.034,0.007] | 0.186 | -0.055 | [-0.070, -0.040] | <0.001 |
| Physical exercise (no as reference) | | | | | | |
| Yes | 0.017 | [-0.001,0.036] | 0.068 | -0.016 | [-0.030, -0.001] | 0.032 |
| Alcohol consumption (no as reference) | | | | | | |
| Yes | 0.002 | [-0.022,0.026] | 0.869 | -0.028 | [-0.047, -0.010] | 0.003 |
| Smoking (no as reference) | | | | | | |
| Yes | -0.026 | [-0.047, -0.004] | 0.021 | -0.010 | [-0.027,0.007] | 0.252 |
| Baseline cognition score (z-score) | 0.740 | [0.731,0.749] | <0.001 | — | | |
| Baseline frailty index (z-score) | — | | | 0.862 | [0.855,0.869] | <0.001 |

**Note:** Models were adjusted for baseline age, gender, educational level, marital status, residence, per capita annual household income, medical insurance coverage, current employment status, physical exercise, alcohol consumption, smoking status, and baseline level of the corresponding outcomes (z-score). **Abbreviations:** Coef, coefficient; CI, confidence interval; G1, Persistently Low Social Activity Engagement Group; G2, Offline Social Activity Dominant Group; G3, Online Social Activity Increasing Group.

**Supplementary Appendix Table S18 | Sensitivity analysis using a restrictive coding of ADL, IADL, and mobility deficits in the frailty index (China, 2011–2018).**

|  | **Frailty progression** | | |
| --- | --- | --- | --- |
|  | Coef | 95% CI | p |
| Time, years | 0.121 | [0.116, 0.127] | <0.001 |
| **Joint online–offline social activity trajectory groups** | | | |
| G1 | Reference |  |  |
| G2 | -0.010 | [-0.025, 0.006] | 0.219 |
| G3 | 0.005 | [-0.029, 0.040] | 0.756 |
| **Joint online–offline social activity trajectory groups × time** | | | |
| G1 × Time | Reference |  |  |
| G2 × Time | -0.020 | [-0.027, -0.013] | <0.001 |
| G3 × Time | -0.049 | [-0.064, -0.034] | <0.001 |
| Age | 0.004 | [0.003, 0.005] | <0.001 |
| Gender (male as reference) | | | |
| Female | 0.029 | [0.012, 0.045] | 0.001 |
| Education level (less than high school as reference) | | | |
| High school or equivalent | 0.013 | [-0.010, 0.036] | 0.252 |
| College and higher | -0.028 | [-0.080, 0.025] | 0.300 |
| Marital status (not married as reference) | | | |
| Married | -0.017 | [-0.038, 0.004] | 0.115 |
| Residence (urban as reference) | | | |
| Rural | 0.006 | [-0.009, 0.020] | 0.430 |
| Per capita annual household income (below the median as reference) | | | |
| At or above the median | -0.012 | [-0.026, 0.002] | 0.083 |
| Medical insurance coverage (uninsured as reference) | | | |
| Insured | -0.013 | [-0.039, 0.012] | 0.308 |
| Current employment status (not currently working as reference) | | | |
| Currently working | -0.050 | [-0.065, -0.035] | <0.001 |
| Physical exercise (no as reference) | | | |
| Yes | -0.011 | [-0.025, 0.003] | 0.135 |
| Alcohol consumption (no as reference) | | | |
| Yes | -0.021 | [-0.040, -0.003] | 0.025 |
| Smoking (no as reference) | | | |
| Yes | -0.006 | [-0.023, 0.011] | 0.479 |
| Baseline restrictive frailty index (z-score) | 0.921 | [0.914, 0.928] | <0.001 |

**Note:** The restrictive frailty index coded ADL, IADL, and mobility items as a deficit only when participants reported that they could not perform the activity. Responses indicating no difficulty, difficulty but can still do it, or difficulty and need help were coded as no deficit. Models were adjusted for baseline age, gender, educational level, marital status, residence, per capita annual household income, medical insurance coverage, current employment status, physical exercise, alcohol consumption, smoking status, and the baseline restrictive frailty index z-score. **Abbreviations:** Coef, coefficient; CI, confidence interval; G1, Persistently Low Social Activity Engagement Group; G2, Offline Social Activity Dominant Group; G3, Online Social Activity Increasing Group.

1. Lennon H, Kelly S, Sperrin M, et al. Framework to construct and interpret latent class trajectory modelling. BMJ Open. 2018;8(7): e020683.
2. Hu Y, Peng W, Ren R, et al. Sarcopenia and mild cognitive impairment among elderly adults: The first longitudinal evidence from CHARLS. J Cachexia Sarcopenia Muscle. 2022;13(6):2944-2952.
